# Dexmedetomidine Attenuates Methotrexate-Induced Neurotoxicity and Memory Deficits in Rats through Improving Hippocampal Neurogenesis: The Role of miR-15a/ROCK-1/ERK1/2/CREB/BDNF Pathway Modulation

**DOI:** 10.3390/ijms24010766

**Published:** 2023-01-01

**Authors:** Mohamed Taha, Omar Mohsen Eldemerdash, Ismail Mohamed Elshaffei, Einas Mohamed Yousef, Mahmoud A. Senousy

**Affiliations:** 1 Department of Biochemistry, Faculty of Pharmacy, Cairo University, Cairo 11562, Egypt; 2 Department of Biochemistry, Faculty of Pharmacy, Misr International University (MIU), Cairo 44971, Egypt; 3 Department of Histology and Cell Biology, Faculty of Medicine, Menoufia University, Shibin el Kom 3251, Egypt; 4 Department of Biochemistry, Faculty of Pharmacy and Drug Technology, Egyptian Chinese University, Cairo 11786, Egypt

**Keywords:** methotrexate, dexmedetomidine, doublecortin, Ki-67, miR-15a, ROCK-1

## Abstract

Methotrexate (MTX) is a widely used neurotoxic drug with broad antineoplastic and immunosuppressant spectra. However, the exact molecular mechanisms by which MTX inhibits hippocampal neurogenesis are yet unclear. Dexmedetomidine (Dex), an α2-adrenergic receptor agonist, has recently shown neuroprotective effects; however, its full mechanism is unexplored. This study investigated the potential of Dex to mitigate MTX-induced neurotoxicity and memory impairment in rats and the possible role of the miR-15a/ROCK-1/ERK1/2/CREB/BDNF pathway. Notably, no former studies have linked this pathway to MTX-induced neurotoxicity. Male Sprague Dawley rats were placed into four groups. Group 1 received saline i.p. daily and i.v. on days 8 and 15. Group 2 received Dex at 10 μg/kg/day i.p. for 30 days. Group 3 received MTX at 75 mg/kg i.v. on days 8 and 15, followed by four i.p. doses of leucovorin at 6 mg/kg after 18 h and 3 mg/kg after 26, 42, and 50 h. Group 4 received MTX and leucovorin as in group 3 and Dex daily dosages as in group 2. Bioinformatic analysis identified the association of miR-15a with ROCK-1/ERK1/2/CREB/BDNF and neurogenesis. MTX lowered hippocampal doublecortin and Ki-67, two markers of neurogenesis. This was associated with the downregulation of miR-15a, upregulation of its target ROCK-1, and reduction in the downstream ERK1/2/CREB/BDNF pathway, along with disturbed hippocampal redox state. Novel object recognition and Morris water maze tests demonstrated the MTX-induced memory deficiencies. Dex co-treatment reversed the MTX-induced behavioral, biochemical, and histological alterations in the rats. These neuroprotective actions could be partly mediated through modulating the miR-15a/ROCK-1/ERK1/2/CREB/BDNF pathway, which enhances hippocampal neurogenesis.

## 1. Introduction

Methotrexate (MTX) is an essential folate antagonist with broad antineoplastic and immunosuppressant spectra. It is indicated, individually or in combination with other drugs, in the treatment of a variety of neoplasia, such as acute lymphoblastic leukemia (ALL), meningeal leukemia, non-Hodgkin lymphoma, osteosarcoma, and uterine, breast, lung, and gestational cancers, as well as in the management of rheumatoid arthritis [1,2]. MTX was recently reported to be one of the most widely used neurotoxic chemotherapeutic agents [3]. MTX-induced neurotoxicity is most often observed in pediatrics during the treatment of ALL, with 9% to 53% of MTX-treated children developing neural deficits [4,5]. However, the incidence and the severity of MTX neurotoxicity are dependent on multiple factors, such as younger age, higher dose, intrathecal route, and combination with radiation [6]. The clinical manifestations of MTX-induced neurotoxicity vary and include convulsion, transient ischemic attacks, encephalopathy, movement disorders, dementia, and deficits in neurocognitive skills [7,8]. Unfortunately, around 50–70% of ALL pediatric survivors suffer from irreversible attention, working memory, and executive function deficits [9].

Although the mechanism of MTX-induced neurotoxicity is not yet well studied, MTX-induced neurotoxicity is attributed to S-adenosylmethionine deficiency, which causes demyelination and folate deficiency. The latter causes impaired DNA synthesis as well as hyperhomocysteinemia, a known causative factor of seizures and vascular diseases resulting in focal neurologic deficits and ischemic white matter changes [10,11]. Furthermore, MTX causes impaired neurogenesis and cell proliferation, survival, and differentiation in the hippocampus in vitro and in vivo [12]. Previous studies have shown that MTX has a neurotoxic effect eliciting memory deficits [13,14], yet the exact pathway underlying this side effect is not clear.

Hippocampus is an area of the brain responsible for working memory and cognitive functions. Hippocampal neurogenesis is an essential process for maintaining memory where new neurons are generated from adult neural stem and progenitor cells in the dentate gyrus (DG) region of the hippocampus [15,16]. Additionally, neurogenesis occurs in the subventricular zone (SVZ) of the lateral ventricle and the subgranular zone (SGZ) of the DG [17]. A growing body of evidence supports that the cyclic AMP (cAMP)-responsive element-binding protein (CREB), brain-derived neurotrophic factor (BDNF), and extracellular signal-regulated kinase 1/2 (ERK1/2) are involved in the regulation of this neurogenesis process [18,19,20]. Activation of CREB is known to cause upregulation of BDNF, which is essential in improving neuronal plasticity, proliferation, differentiation, and survival in the hippocampus as well as enhancing cognitive functions, learning, and memory abilities [21,22]. In addition, evidence indicates that ERK1/2 plays an important role in gene regulation through chromatin remodeling as well as phosphorylation and activation of CREB, thus instigating the neurogenesis process [20]. However, the effect of MTX on the ERK1/2/CREB/BDNF pathway as well as doublecortin (DCX), a marker for immature neurons, is not well identified.

MicroRNAs (miRNAs), a class of non-coding RNAs, play a fundamental role in post-transcriptional gene expression regulation. Neural miRNAs are essential components of the gene regulatory networks that govern the multistep process of adult neurogenesis [23]. Upstream of the ERK1/2/CREB/BDNF signaling pathway, miR-15a modulates gene expression and has been lately linked to memory regulation and neurogenesis. miR-15a was reported to be downregulated in temporal lobe epilepsy and Alzheimer’s disease in human and mice models [24,25,26]. Moreover, miR-15a targets and represses Rho-associated protein kinase 1 (ROCK-1), a well-known inhibitor of ERK1/2, with subsequent activation of ERK1/2/CREB, resulting in increased cell viability and limited apoptosis [27,28].

Dexmedetomidine (Dex) is a powerful agonist of the α2-adrenergic receptor with sedative, analgesic, and anxiolytic properties that is safe to use in children [29]. Recently, Dex was reported to exert a neuroprotective effect and attenuate propofol-induced neurotoxicity in hippocampal cell lines and hyperoxia-induced toxicity in neonatal rat brains [30,31]. It also exerted a neuroprotective effect on chemotherapy-induced cognitive impairment [32], making it a promising candidate for attenuating MTX-induced toxic effect. Dex was also found to exert a neuroprotective effect by promoting neurogenesis through CREB activation as well as binding to imidazoline I_1_ receptor and modulating histone acetylation via ERK1/2 pathways [33,34]. Lately, Dex was reported to attenuate cisplatin-induced cognitive impairment in rats through modulating the expression of miR-429-3p [35] and was proposed as a possible treatment for MTX-induced neurotoxicity and inflammation in hippocampal HT22 cell lines [36]. However, to the best of our knowledge, the mechanisms that underpin the neuroprotective effect of Dex and its ability to attenuate MTX-induced neurotoxicity have not been previously studied in experimental models. More specifically, the effects of MTX and Dex on the miR-15a/ROCK-1/ERK1/2/CREB/BDNF signaling pathway remain unexplored.

Therefore, this study investigated the role of the miR-15a/ROCK-1/ERK1/2/CREB/BDNF pathway in MTX-induced neurotoxicity and memory deficits in rats. Furthermore, we sought to assess the potential biochemical, molecular, and cellular mechanisms of Dex against MTX-induced memory deficits and impaired hippocampal neurogenesis. Our study provides novel evidence that Dex co-treatment alleviated the MTX-induced behavioral and biochemical changes, highlighting its neuroprotective actions through modulating the miR-15a/ROCK-1/ERK1/2/CREB/BDNF signaling pathway.

## 2. Results

### 2.1. Dex Improves the Behavioral and Memory Deficits Induced by MTX Administration

To assess the effects of MTX and Dex co-treatment on behavioral and memory changes, we conducted the novel object recognition (NOR) and the Morris Water Maze (MWM) tests.

**Figure 1:**
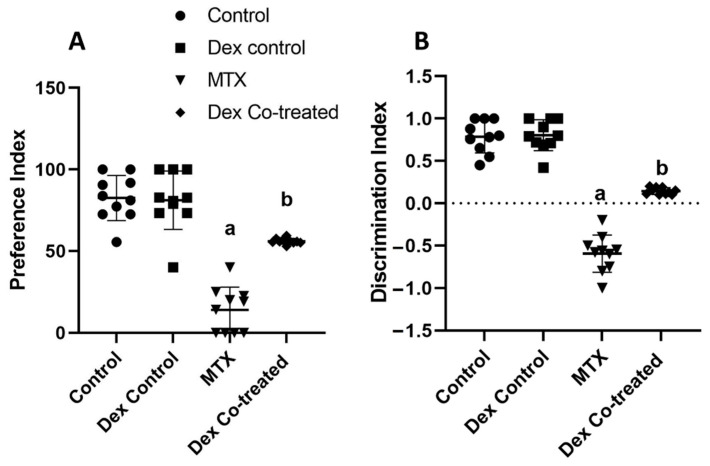
Cognitive and behavioral effects of MTX and Dex co-treatment (novel object recognition test). (**A**) Preference index for the novel object recognition test indicates a lower preference for the novel object in the MTX group compared to other groups. (**B**) Discrimination index for the novel object recognition test indicates negative discrimination between the novel and familiar objects in the MTX group compared to other groups. Data are expressed as mean ± SD, *n* = 10. (a) Significant difference from control, and (b) significant difference from MTX. Significance level is set at *p* < 0.05. One-way ANOVA followed by Tukey’s post hoc test were used in the statistical analysis.

The NOR test determines the ability of rats to remember familiar objects and recognize novel one through measuring two indices: the preference index and the discrimination index. The preference index (PI) was calculated by dividing the time spent investigating the novel object by the total time spent investigating both novel and familiar objects and then multiplying by 100. The PI significantly decreased by 82.49% in the MTX-intoxicated group compared with that in the normal control group. However, Dex co-treatment meaningfully increased the PI by 3.9-fold, returning it near the normal level (*p* < 0.05) (Figure 1A). The discrimination index (DI) was calculated by dividing the difference between the time spent examining a novel object and the time spent exploring a familiar object by the time spent exploring both novel and familiar objects. The MTX treatment inverted the ratio of the DI, resulting in a 175.49% decrease in DI relative to normal. Co-treatment with Dex restored the DI to a positive value and increased the DI by 1.73-fold (*p* < 0.05) (Figure 1B). These findings indicate that MTX administration impairs spatial memory, whereas Dex co-treatment is effective in reducing this impairment.

**Figure 2:**
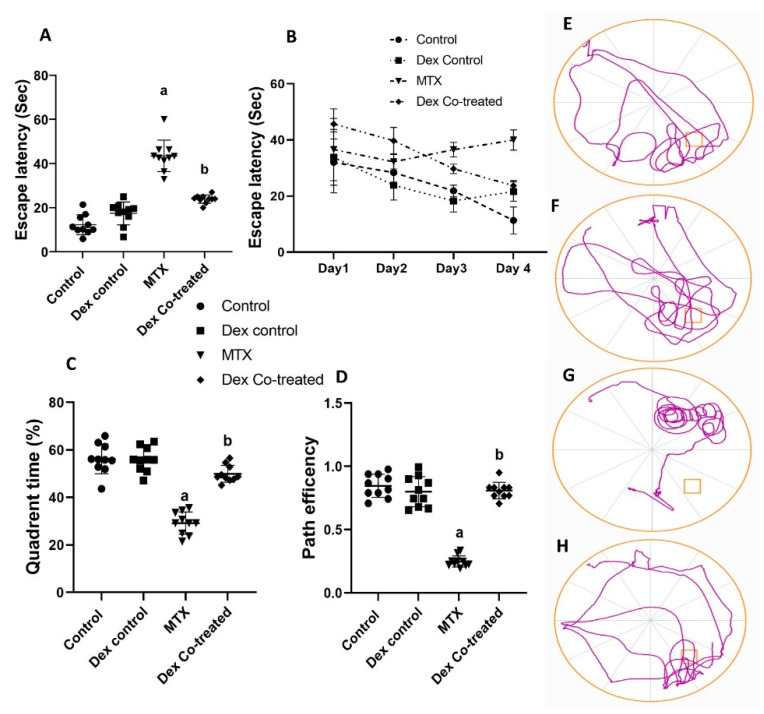
Cognitive and behavioral effects of MTX and Dex co-treatment (Morris water maze test). (**A**) Escape latency for the Morris water maze in the probe test indicating higher escape latency in the MTX group. (**B**) Escape latency of the training trials and probe test indicating lower pattern of spatial memory in the MTX group. (**C**) Quadrant time indicates that the time percentage a rat stayed in the targeted quadrant in the probe test is lower in the MTX-treated rats. (**D**) Path efficiency indicating lower path accuracy in MTX group. (**E**) Track plot of a rat from the control group. (**F**) Track plot of a rat from the Dex control group. (**G**) Track plot of a rat from the MTX group. (**H**) Track plot of a rat from the Dex co-treated group. Data are expressed as mean ± SD, *n* = 10. (a) Significant difference from control, and (b) significant difference from MTX. Significance level is set at *p* < 0.05. One-way ANOVA followed by Tukey’s post hoc test were used in the statistical analysis, except for the training and probe escape latency where two-way ANOVA was used.

Next, we investigated the learning ability and memory function of the rats in different experimental groups by measuring the escape latency (the time to reach the hidden platform) from the MWM. Our results showed that the rats in the MTX group took significantly longer time to reach the platform area by 3.79-fold more than the normal control rats (*p* < 0.05), indicating memory deficit. On the other hand, Dex co-treatment significantly increased the learning ability of the rats, with a 45.25% reduction in the escape latency time compared to that in the MTX group (*p* < 0.05) (Figure 2A). Furthermore, investigating the escape latency during the training trials and the probe test, which studied the learning pattern of the rats, revealed that the MTX group had lower learning ability and decreased escape latency progress over days than the control and the Dex co-treated groups (Figure 2B). In line with these results, the quadrant time percentage (time percentage in which the rat remained in the target quadrant in the probe test) was lower in the MTX group by 52% compared to that in the normal control rats. Interestingly, the quadrant time percentage increased by almost 2-fold in the Dex co-treated group compared to that in the MTX group (*p* < 0.05) (Figure 2C). Furthermore, we detected path efficiency, which is the ratio between the actual path length traveled by a rat and the ideal path it might have taken to reach the target quadrant. The MTX group showed a marked decrease in path accuracy by 59.94% compared to the normal rats, whereas the rats co-treated with Dex showed a significant improvement, as depicted in a 3.26-fold increase in path efficiency compared to that in the MTX group (*p* < 0.05) (Figure 2D). The path taken by the rats was detected and plotted in the track plots, which obviously indicate more quadrant time in the Dex co-treated group than the MTX group (Figure 2E–H).

### 2.2. Possible Molecular Mechanisms Underlie the Dex Effects on the Neurogenesis Process in MTX-Treated Rats

#### 2.2.1. Dex Restores the BDNF Downregulation Induced by MTX

**Figure 3:**
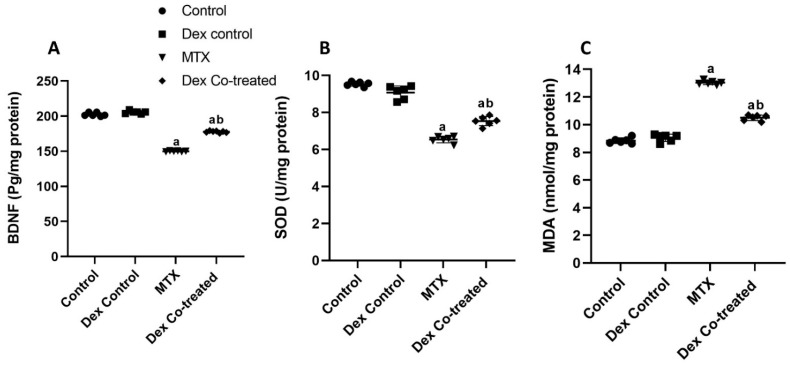
Effects of MTX and Dex co-treatment on expression of BDNF and oxidative stress markers. (**A**) MTX induces a significant decrease in BDNF expression level when compared to the normal control. In addition, a significant increase in BDNF expression is detected in the Dex-cotreated group when compared to the MTX-treated rats. (**B**,**C**) MTX administration induces a significant decrease in SOD and a significant increase in MDA levels compared to the normal control rats. This effect is reversed upon Dex co-treatment as a significant increase in SOD and a significant decrease in MDA levels are detected when compared to the MTX-treated group. Data are expressed as mean ± SD, *n* = 6. (a) Significant difference from control, and (b) significant difference from MTX. Significance level is set at *p* < 0.05. One-way ANOVA and Tukey’s post hoc test were used in the statistical analysis.

Next, to study the various mechanisms underlying the neuroprotective effect of Dex against MTX-induced neurotoxicity, we assessed the expression of BDNF, a neurotrophic protein that is crucial for neuronal survival and synaptic plasticity. Our results showed that MTX induced a significant decrease in BDNF expression level by 25.96% compared to that in the normal control rats (*p* < 0.05) (Figure 3A). Moreover, a significant increase in BDNF expression by 24.48% was detected in the Dex co-treated group when compared to that in the MTX treated rats (*p* < 0.05) (Figure 3A). Together, these results show that Dex administration markedly restored the BDNF downregulation induced by MTX.

#### 2.2.2. Dex Alleviates MTX-Induced Oxidative Stress in the Hippocampal Tissues of Rats

To further understand the effects of Dex on the neurogenesis process of the MTX-treated rats, we investigated the levels of two oxidative stress markers, superoxide dismutase (SOD) and malondialdehyde (MDA). Our results indicated that MTX administration increased oxidative stress as evidenced by a significant decrease in SOD by 31.48% and an increase in MDA level by 47.45%, compared to the levels in the normal control rats (*p* < 0.05) (Figure 3B,C). In contrast, Dex co-treatment significantly decreased the oxidative stress status as shown by an increase in the SOD level by 15.28% (*p* < 0.05) and a decrease in the MDA level by 19.4%, compared to the levels in the MTX-treated group (*p* < 0.05) (Figure 3B,C). Our findings indicate the ability of Dex to reduce MTX-induced oxidative stress in hippocampal tissues.

#### 2.2.3. miR-15a Promotes Neurogenesis via Regulating ROCK-1/ERK1/2/CREB/BDNF Transcription Factors: Extensive Bioinformatic Approach

**Figure 4:**
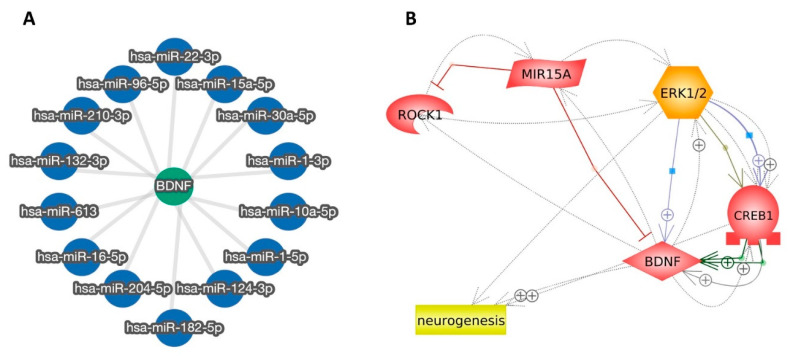
miR-15a promotes neurogenesis via regulating ROCK-1/ERK1/2/CREB/BDNF transcription factors: Bioinformatic analyses. (**A**) An interaction graph created by the miRTargetLink 2.0 tool showing miRNAs that target and, more specifically, modulate the BDNF proneuronal transcription factor activities. All the included miRNA–BDNF interactions are strongly validated and functional. (**B**) Network analysis of miR-15a, BDNF, ROCK-1, ERK1/2, CREB, and neurogenesis. The generated network demonstrates that miR-15a directly inhibits ROCK-1 and stimulates ERK1/2. No direct association is detected between miR-15a and CREB, however, activated ERK1/2 has positive regulatory effects on CREB and BDNF that subsequently enhance the neurogenesis process. Of note, the detected miR15a inhibitory action on BDNF is based on studies conducted on hepatocellular and hematological malignancies.

We employed the miRTargetLink 2.0 interactive tool (https://ccb-compute.cs.uni-saarland.de/mirtargetlink2/network/bf99b12a-cd13-453e-bcc1-aa4226ac943e, accessed on 19 Dec 2021) to detect the miRNAs that target and regulate the proneuronal transcription factor BDNF. Our bioinformatic analysis revealed the miRNAs (miR-1, miR-10a, miR-124, miR-132, miR-15a, miR-16, miR-182, miR-204, miR-210, miR-22, miR-30a, miR-613, and miR-96) that more specifically modulate the BDNF transcription factor activities. All the detected miRNAs-BDNF interactions are strongly validated and functional based on different experimental approaches (Supplementary File S1 and Figure 4A). After carefully reviewing the literature and using the Human microRNA Disease Database (HMDD) (https://www.cuilab.cn/hmdd, accessed on 9 Jan 2022), we detected an association of miR-15a with the pathogenesis of many neurological disorders, such as Alzheimer’s Disease, epilepsy, and multiple sclerosis.

Next, we decided to further analyze the possible associations between miR-15a and BDNF using the freely accessible bioinformatic tool by Pathway Studio (https://www.pathwaystudio.com, accessed on 9 January 2022). We included in our analysis ROCK-1, ERK1/2, and CREB transcription factors, which are known to be involved in the regulation of the neurogenesis process associated with BDNF. As depicted in (Figure 4B), complex associations between miR-15a and the previously mentioned transcription factors were detected. Our bioinformatic findings demonstrate that miR-15a directly inhibits ROCK-1 and stimulates ERK1/2. Of note, the detected miR15a inhibitory action on BDNF is based on studies conducted on hepatocellular and hematological malignancies [37,38]. No direct association was detected between miR-15a and CREB; however, activated ERK1/2 was detected to have positive regulatory effects on CREB and BDNF that subsequently enhance the neurogenesis process. Our bioinformatic data collectively demonstrate that miR-15a activates ERK1/2, CREB1, and BDNF with subsequent induction of neurogenesis.

#### 2.2.4. Modulation of miR-15a/ROCK-1/ERK1/2/CREB Signaling Pathway by Dex in MTX-Treated Rats

**Figure 5:**
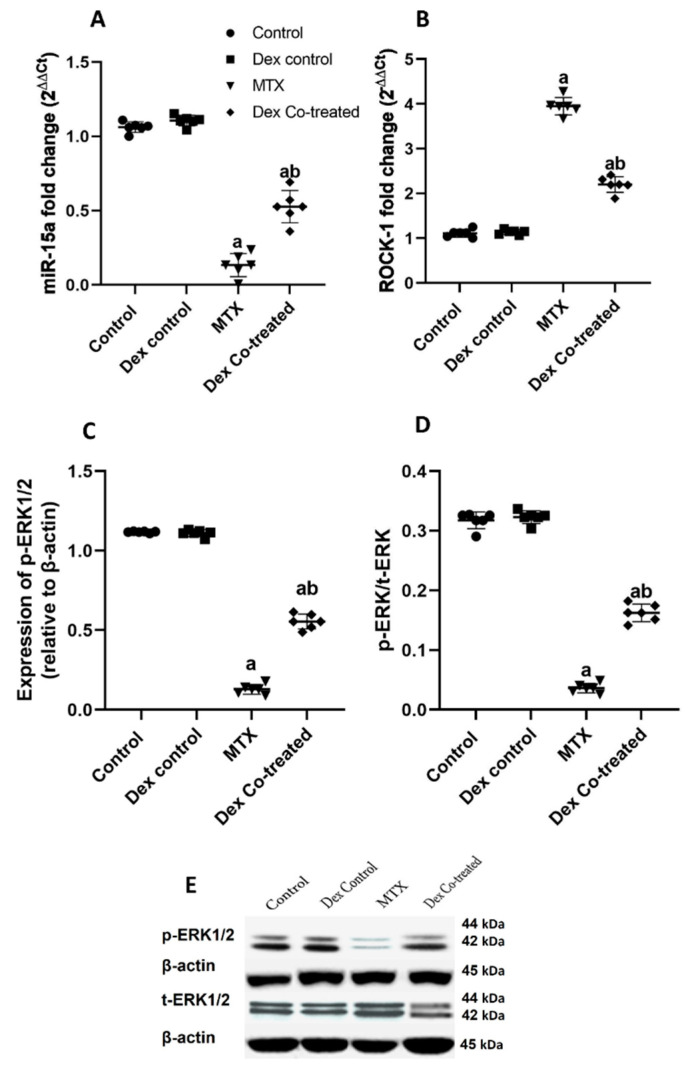
Effects of MTX and Dex on miR-15a, ROCK-1, and ERK1/2. (**A**,**B**) The MTX-treated group has a significant decrease in miR-15a and an increase in ROCK-1 expression compared to the normal control group. However, Dex co-treatment significantly reverses the MTX effect by significantly increasing miR-15a and lowering ROCK-1 expression levels when compared to the MTX-treated group. (**C**,**D**) In comparison to the normal control group, the MTX-treated rats show significant decreases in p-ERK and the ratio of p-ERK to t-ERK. On the other hand, Dex co-treatment restores ERK1/2 activation as demonstrated by a rise in p-ERK as well as in the p-ERK/t-ERK ratio, compared to the MTX group. (**E**) Representative Western blot of the expression levels of p-ERK and t-ERK. Data are expressed as mean ± SD, *n* = 6. (a) Significant difference from control, and (b) significant difference from MTX. Significance level is set at *p* < 0.05. One-way ANOVA and Tukey’s post hoc test were used in the statistical analysis.

Based on the findings of our bioinformatic analysis and a comprehensive review of the literature, we sought to further investigate the possible involvement of miR-15a and ROCK-1/ERK1/2/ CREB/BDNF in MTX-induced neurotoxicity and the neuroprotective effects of Dex in different groups of our experimental model. Our results demonstrated that the MTX-treated group had a significant decrease in miR-15a expression by 87.4% and a 3.5-fold increase in ROCK-1 expression level compared to those in the normal control rats (*p* < 0.05) (Figure 5A,B). These findings are consistent with our bioinformatic analysis which shows that miR-15a negatively regulates ROCK-1. The present qRT-PCR results showed that Dex co-treatment significantly reversed the MTX effect as shown in the increased miR-15a expression level by 3.93-fold and the lowered ROCK-1 expression level by 44.26 %, compared to the levels in the MTX-treated group (*p* < 0.05) (Figure 5A,B).

**Figure 6:**
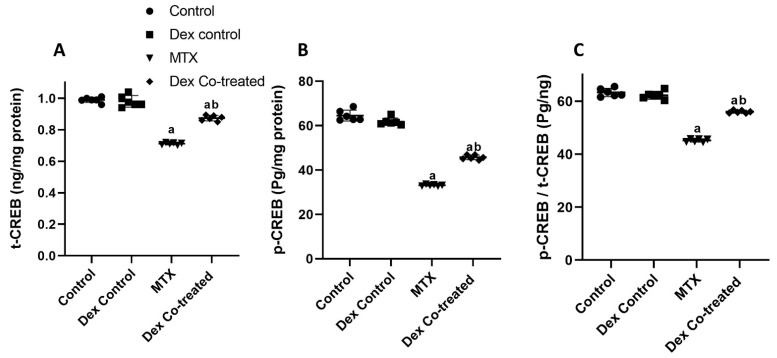
Effect of MTX and Dex on CREB expression and activation. MTX administration induces significant decrease in the expression of t-CREB (**A**), p-CREB (**B**), and p-CREB/t-CREB ratio (**C**) when compared to the normal control. However, Dex increases the expression of t-CREB (**A**), p-CREB (**B**), and p-CREB/t-CREB ratio (**C**) compared to the MTX group. Data are expressed as mean ± SD, *n* = 6. (a) Significant difference from control, and (b) significant difference from MTX. Significance level is set at *p* < 0.05. One-way ANOVA and Tukey’s post hoc test were used in the statistical analysis.

As ROCK-1 is well established as an inhibitor of the ERK1/2/CREB signaling pathway, we sought to assess the total and phosphorylated forms of these transcription factors in our experimental rat model. In comparison to the normal control group, the MTX-treated rats showed significant decline in ERK1/2 activation as evidenced by a decrease in phosphorylated ERK1/2 (p-ERK) by 88.45%, where the ratio of p-ERK1/2 to total ERK1/2 (t-ERK1/2) declined by 88.52% (*p* < 0.05) (Figure 5C–E). Dex co-treatment, on the other hand, restored ERK1/2 activation, which had been severely suppressed by MTX, as demonstrated by a 4.15-fold rise in p-ERK level compared to that in the MTX group, along with a 4.18-fold increase in the p-ERK/t-ERK ratio (*p* < 0.05) (Figure 5C–E). We also detected that MTX administration induced significant decrease in the expression levels of total CREB (t-CREB), phosphorylated CREB (p-CREB), and p-CREB/t-CREB ratio by 22.49%, 44.54%, and 26.47%, respectively, when compared to those in the normal control rats (*p* < 0.05). By contrast, our results revealed the ability of Dex to increase the expression levels of t-CREB, p-CREB, and p-CREB/t-CREB ratio by 22.8%, 37.93%, and 23.99%, respectively, compared to levels in the MTX group (*p* < 0.05) (Figure 6A-C).

### 2.3. Possible Cellular Mechanisms Underpin the Dex Effects on the Neurogenesis Process in MTX-Treated Rats

#### 2.3.1. Dex Preserves Hippocampal Neurons from MTX-Induced Neuronal Injury

**Figure 7:**
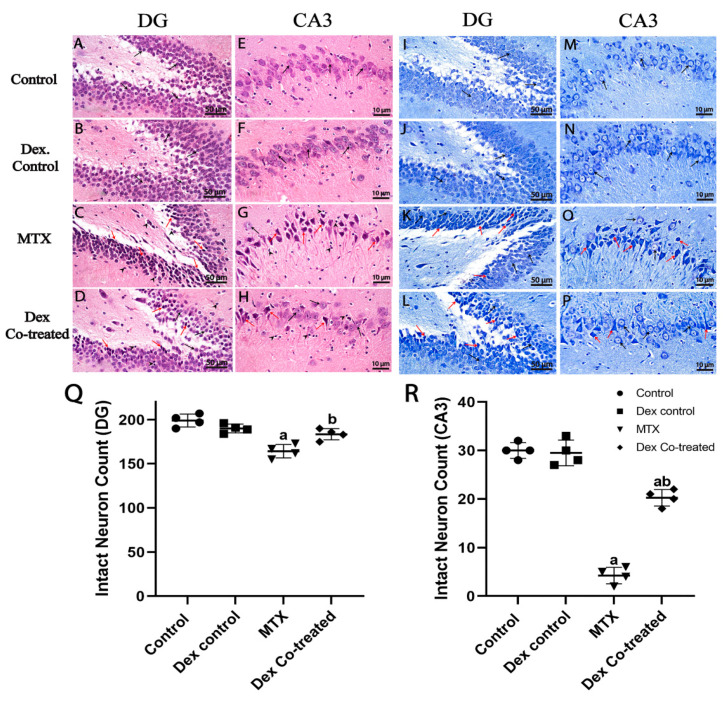
Hematoxylin and Eosin (H&E) and Nissl staining of hippocampus. (**A**–**H**) H&E staining, and (**A**,**B**) normal morphology with intact subcellular details (arrow). (**C**) shows higher degeneration of granule neurons with higher figures of nuclear pyknosis (red arrow) and higher reactive glial cell infiltrates (arrowhead). (**D**) shows almost the same records as MTX with mildly higher records of apparent intact granule cells. (**E**,**F**) normal morphology with intact well-organized pyramidal neurons and intact nuclear and subcellular details (black arrow). (**G**) severe neuronal loss and hypereosinophilic, angular necrotic pyramidal neurons without distinct subcellular details (red arrow) alternated with few scattered apparent intact cells (black arrow) and markedly higher-reactive microglial cell infiltrates (arrowhead). (**H**) moderate protective efficacy with persistent records of damaged and necrotic neurons (red arrow) alternated with higher records of apparent intact neurons (black arrow) and milder-reactive glial cell infiltrates (arrowhead). (**I–P**) Nissl staining showing intact neurons (black arrow) and damaged neurons (red arrow). (**Q**) Intact neuron count in DG. (**R**) Intact neuron count in CA3. Data are expressed as mean ± SD, *n* = 4. (a) Significant difference from control, and (b) significant difference from MTX. Significance level is set at *p* < 0.05. One-way ANOVA with Tukey’s post hoc test were used in the statistical analysis. (A,D) H&E 400×, (E,H) H&E 1000×, (I,L) Nissl staining 400×, (M,P) Nissl staining 1000×.

A light microscopic examination of the hippocampal DG regions from various Hematoxylin and Eosin (H&E)-stained sections revealed that both normal and Dex control groups had the characteristic morphological features of hippocampal layers, including granule cells at various zones with intact subcellular details as well as a hilar region without abnormal alterations. The MTX-treated group showed marked change in the DG in the form of increased degenerated granule neurons with higher figures of nuclear pyknosis, moderate edema, and mildly higher-reactive glial cell infiltrates. The Dex co-treated groups exhibited improvement in the hippocampal DG region in the form of preservation of intact granule cells; however, some pyknotic granule cells together with reactive glial cell infiltrates were detected (Figure 7A–D).

A histological examination of the Cornu Ammonis 3 (CA3) area in both normal and Dex control groups indicated normal hippocampal layer morphology with intact well-organized large pyramidal neurons with intact nuclear and subcellular features, and an intact intercellular matrix was observed with minimally reactive glial cell infiltrates. Administration of MTX induced severe neuronal loss together with a marked shrinkage of large pyramidal neurons (hypereosinophilic, angular necrotic, and indistinct subcellular details). Few scattered apparent intact cells and mild perineuronal edema were also detected in the brain matrix with markedly higher-reactive microglial cell infiltrates. Co-treatment with Dex preserved the large pyramidal neurons when compared to those in the MTX-treated group, indicating its protective efficacy. However, few damaged and necrotic neurons, as well as mildly reactive glial cell infiltrates, were also detected (Figure 7E–H).

We further employed Nissl staining to detect degraded and intact neuron count in the DG and CA3 hippocampal regions from all experimental groups (Figure 7I–P). All cells were counted in these two regions using a light microscope at 400X magnification. The MTX-treated rats showed a significant decrease in intact granule cells by nearly 16% in the DG region and a dramatic decrease in the large pyramidal cells’ density in the CA3 region by nearly 85.5%, compared to the normal control. By contrast, the quantity of intact neurons in the DG area was significantly elevated in the Dex co-treatment group by 12.63% compared to that in the MTX-treated group, which was comparable to the normal control (*p* < 0.05) (Figure 7Q). Furthermore, a significantly high fold change (4.65-fold) in the intact large pyramidal neuron count was detected in the CA3 region after co-treatment with Dex compared to that in the MTX-treated group (*p* < 0.05) (Figure 7R). These results suggest the potential neuroprotective effects of Dex against MTX- induced reduction in the hippocampal neurons in adult rats.

#### 2.3.2. Dex Increases the Number of Immature Neurons in the Hippocampus of Rats Treated with MTX

**Figure 8:**
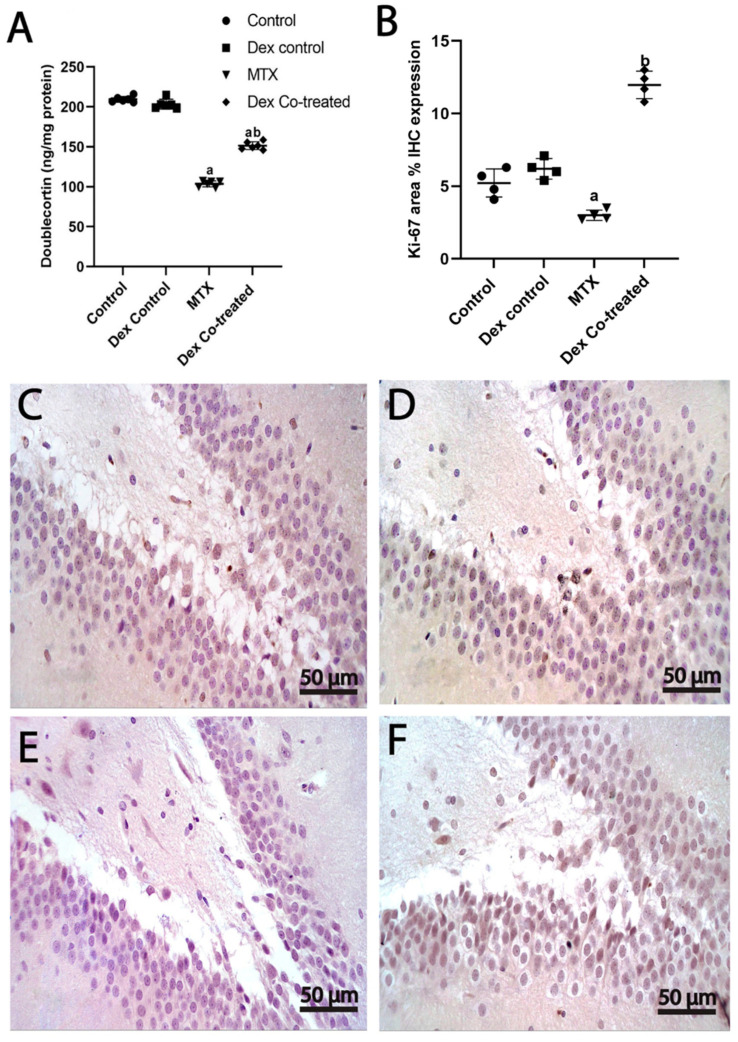
Effect of MTX and Dex on the number of immature neurons and cell proliferation. (**A**) The expression of DCX significantly decreases in the MTX-treated rats compared to the normal control rats; however, DCX expression significantly increases in the Dex co-treated groups when compared to the MTX-treated group, (**B**) Area-based % of Ki-67 immunohistochemical expression: Ki-67 expression is significantly lower in the MTX-treated group compared to the normal control group. Dex administration increases the % of Ki-67 positive cells when compared to the MTX-treated group. Ki-67 immunohistochemical staining of (**C**) normal control, (**D**) Dex control, (**E**) MTX, and (**F**) Dex co-treated group. Data are expressed as mean ± SD, *n* = 4 for Ki-67 immunohistochemical staining where *n* = 6 for DCX. (a) Significant difference from control, and (b) significant difference from MTX. Significance level is set at *p* < 0.05. One-way ANOVA with Tukey’s post hoc test were used in the statistical analysis. (**C**,**F**) Ki-67-stained sections 400×).

We used doublecortin (DCX) expression analysis to assess the count of hippocampal immature neurons. The expression of doublecortin significantly decreased by 50.5% in the MTX-treated rats compared to that in the normal control rats (*p* < 0.05), which indicates that MTX may negatively affect neurogenesis in the hippocampus. In contrast, the expression level of DCX was noticed to significantly increase in the Dex co-treated group by 1.45-fold compared to that in the MTX-treated group (*p* < 0.05). These findings suggest that Dex is able to counteract the MTX-induced loss of immature neurons, indicating an increase in neurogenesis (Figure 8A).

#### 2.3.3. Dex Promotes Cell Proliferation in the Hippocampus of Rats Treated with MTX

Ki-67 immunohistochemical staining was used to evaluate cell proliferation in the SGZ of the hippocampal DG region by estimating the area-based percentage of Ki-67 positive cells. Overall, the percentage of Ki-67 positive cells varied significantly between our experimental groups (*p* < 0.05). The percentage of Ki-67 positive cells in the rats treated with MTX was 44.27% lower than that in the control group (*p* < 0.05). In contrast, the rats co-treated with Dex showed 2.3-fold and 4.18-fold elevated levels of the percentage of Ki-67 positive cells in comparison to the levels in the normal control and the MTX-treated groups, respectively (*p* < 0.05) (Figure 8B–F).

## 3. Discussion

To the best of our knowledge, this study is the first to examine the whole molecular pathway of the neurotoxic effect of MTX and to provide in vivo evidence that Dex effectively modulates MTX-induced neurotoxicity in rat hippocampus. DEX, a highly selective α2- adrenergic agonist, has shown interesting anti-inflammatory, cardioprotective, and neuroprotective properties [39]. Our pilot study data highlight the importance of the use of leucovorin (LCV) combined with MTX in the clinical setting to reduce other systemic side effects. In this study, we also demonstrated that Dex has neuroprotective actions against MTX-induced neurotoxicity that could be possibly through modulating the hippocampal miR-15a/ROCK-1/ERK1/2/CREB/BDNF neurotropic signaling pathway.

We performed both NOR and MWM tests to assess the cognitive function of rats, including learning ability and memory. Our results demonstrated that MTX treatment induced a marked decline in the rats’ cognitive function as they did not show any preference to the novel object. Moreover, the MTX-treated rats took longer time to reach the platform area by 3.79-fold more than the normal rats and showed 59.94% decrease in path efficiency, indicating cognitive and memory deficits caused by MTX-induced neurotoxicity. Our findings are consistent with previously published studies that reported a decline in hippocampal neurogenesis, resulting in memory deficits and cognitive impairment following MTX administration [13,14,40]. Obviously, it is well known that MTX adversely affects other brain regions, including the cerebellum and cerebral cortex, causing various neurotoxic effects and leukoencephalopathy [41,42,43]. However, these regions are not widely known to be implicated in memory and learning behaviors. Furthermore, hippocampus is most linked to learning and memory [44]. The suggested mechanisms involved in the MTX-induced neurotoxicity may be attributed to a reduction of intracellular adenosylmethionine and folate, resulting in demyelination and defective DNA, RNA, and protein syntheses. In addition, MTX is known to induce hyperhomocysteinemia, a known causative factor of seizures and vascular diseases, resulting in focal neurologic deficits and ischemic white matter changes [10,11]. These mechanisms could explain how MTX causes impaired hippocampal neurogenesis and reduction in cell proliferation, survival, and differentiation [12]. Interestingly, Dex co-treatment with MTX showed significant improvement in both PI and DI in the NOR tests as well as escape latency, quadrant time, and path efficiency in the MWM tests. Our results from these behavioral tests proved the positive effect of Dex on restoring the behavioral and cognitive impairment induced by MTX administration. Our results highlighted the benefits of using Dex co-treatment with MTX and other neurotoxic medications.

We further explored the possible mechanisms by which MTX and Dex induce their effects on the rats’ hippocampus. Our findings demonstrated that MTX administration is associated with a substantial decrease in BDNF expression when compared to the normal control. BDNF is a neurotrophic protein that plays an influential role in neural survival and synaptic plasticity and exerts its effect via a variety of mechanisms and regulations. It has been reported that BDNF specifically binds to tropomyosin receptor kinase B (TrkB) and fosters cell survival through its downstream signaling pathway [45,46]. In addition, the neuroprotective effects of BDNF have been associated with decreased reactive oxygen species production [47]. Both the decrease in BDNF and the increase in oxidative stress have been considered as common causes for neurodegenerative disorders, including cognitive dysfunction, Parkinsonism, Huntington’s disease, and schizophrenia [48,49,50,51]. This is supported by our findings which indicate that MTX administration is associated with oxidative stress as evidenced by increased MDA along with decreased SOD in the rats’ hippocampal tissues in comparison to the normal control. Notably, some studies support the positive relation between BDNF expression, neurogenesis, and cell proliferation [50,52], while other research has denied this hypothesis [53]. On the other hand, Dex co-treatment elevated the BDNF expression and reversed the MTX-induced oxidative stress, as evidenced by the escalated SOD, and decreased MDA levels in the Dex co-treated group when compared to the MTX-treated rats. Similarly, previous studies have reported that Dex is able to alleviate oxidative stress injury in the vascular smooth muscle and lungs [54,55,56]. Together, our results reveal that Dex administration reduces the neurotoxic effect of MTX via upregulating BDNF expression and reducing oxidative stress in rats’ hippocampus.

Next, we decided to explore potential genetic and epigenetic factors which regulate the proneuronal transcription factor BDNF using multi-approach bioinformatic analyses and extensive searching of the literature. Our results demonstrated an association of miR-15a with the pathogenesis of many neurological disorders, such as Alzheimer’s disease, epilepsy, and multiple sclerosis. This is in line with previous studies which have reported downregulation of miR-15a is in Alzheimer’s disease [24,27]. Our bioinformatic analysis using Pathway Studio detected complex associations between miR-15a, ROCK-1, ERK1/2, and CREB transcription factors, which are known to be involved in the regulation of the neurogenesis process and are associated with BDNF. We demonstrated that miR-15a directly inhibits ROCK-1 and stimulates ERK1/2. We could not detect any direct association between miR-15a and CREB; however, activated ERK1/2 was detected to have positive regulatory effects on CREB and BDNF with subsequent enhancement of neurogenesis. Our bioinformatic data collectively demonstrate that miR-15a promotes neurogenesis via regulating ROCK-1/ERK1/2/CREB/BDNF transcription factors.

We used RT-qPCR, western blot, and other biochemical assays to validate the bioinformatic findings in our experimental rat model. Consistent with our bioinformatic analyses, the MTX-treated group exhibited a substantial decrease in hippocampal miR-15a expression, an increase in ROCK-1 expression, and a decline in ERK1/2 activation, as evidenced by a decrease in p-ERK and the ratio of p-ERK to t-ERK, when compared to the normal control. These findings support prior studies which have demonstrated ROCK-1/ERK1/2 inhibitory crosstalk by reporting increased ERK activity in ROCK-inhibitor treated models [57,58]. Indeed, inhibition of ROCK-1 activates ERK1/2, favoring the adipogenesis process [59].

We also detected that MTX administration induced considerable reduction in the expression of t-CREB, p-CREB, and p-CREB/t-CREB. CREB is a transcriptional factor that binds to cAMP response element (CRE), activitaing the expression of some important genes, such as BDNF [60,61]. Our results are in agreement with those of Zhang et al. who related elevated ROCK-1 and downregulated ERK1/2/CREB/BDNF expression levels with increased oxidative stress and apoptosis in retinal Müller cells [62]. The observed decrease in CREB expression and activation in our experiment can be attributed to ROCK-1 overexpression and decreased ERK1/2 activation, which are crucial for CREB phosphorylation [20]. On the other hand, Dex exerted a stimulatory effect on the hippocampal ERK1/2/CREB/BDNF pathway in the MTX-treated rats. This can be supported by a study that has shown that the neuroprotective effect of Dex was abolished in all groups using PD98059, H89, and KG501 as the inhibitors for ERK1/2 and CREB [30]. Furthermore, the effect of Dex on the I1 receptor was found to be crucial for its effect on ERK1/2 and its phosphorylation. Previously published data have highlighted the agonistic effect of Dex on I1 receptor as a p-ERK inducer that is diminished when I1 receptor antagonist is used [63].

Herein, Dex co-treatment reversed the MTX effects, as evidenced by escalated miR-15a expression, ROCK-1 downregulation, restored ERK1/2 activation, and increased expression of t-CREB, p-CREB, and p-CREB/t-CREB. These results also support the findings of a prior study which has reported that elevated miR-15a expression improves the spatial learning and memory abilities in a mice model with Alzheimer’s disease [27]. Moreover, our findings are consistent with previous observations by others who have proved the anti-inflammatory effect of Dex through ROCK-1 inhibition in cardiac muscle cells and NCM460 cells [64,65]. Taken together, these results suggest that Dex administration reduces the neurotoxic effect of MTX via modulating the miR-15a/ROCK-1/ERK1/2/CREB/BDNF signaling pathway.

The hippocampus is composed of different areas, including DG and CA which is subdivided into CA1, CA2, CA3, and CA4 regions [66,67]. Hippocampal neurogenesis in the DG and the mossy fiber pathway between the DG and CA3 plays an important role in memory process [66]. So, we decided to assess the effects of MTX and Dex on the histological features of the hippocampus of different experimental groups in this model.

In this study, the MTX-treated group showed significant changes in the DG and CA3 areas of hippocampus, including severe neuronal loss together with a marked shrinkage of large pyramidal neurons. Immunohistochemical analysis demonstrated that Ki-67 positive cells markedly decreased in the MTX group in both DG and CA3 areas. Additionally, the level of DCX, an essential factor for the neurogenesis process and a marker of immature neurons, was reduced to half of its normal values in the MTX group. Thus, our findings suggest that MTX-induced neurotoxicity might be the consequence of diminished neurogenesis, cell proliferation, and cell survival. Importantly, these findings could explain the behavioral changes detected in the rats of the MTX-treated group. This is consistent with the findings of previous studies which have demonstrated that depressed hippocampal neurogenesis is known to lower the performance of rats in any hippocampal related duties as learning and memory deficits [13,40].

Intriguingly, Dex co-treatment preserved the DG and CA3 hippocampal intact granule cells and the large pyramidal neurons in the MTX-treated rats. Moreover, our findings prove that Dex increases cell proliferation and the number of immature neurons, as evidenced by an increase in the number of Ki-67 and DCX positive cells in the hippocampus of rats treated with MTX. Taken together, these results imply that Dex is a neurogenesis activator that can be used in combination with MTX throughout the therapy regimen to provide neuroprotection.

### Limitations of the Study

Our study is limited by the lack of data on the effects of MTX and Dex on other regions of the brain other than the hippocampus. Further work is needed to cover this point in order to find more conclusive answers. Although we did not observe any significant differences between the Dex control group and the normal control rats regarding all the studied parameters, other organ toxicities that might be provoked by this drug or its dosage should be further evaluated. We also urge additional research on other molecular pathways that may be altered by MTX to determine whether they are related or not.

## 4. Materials and Methods

### 4.1. Experimental Animals

Sixty male Sprague Dawley rats, weighing 150–200 g and aged 4–5 weeks, were obtained. The animals were housed in a controlled environment at the Misr International University’s animal house, with a consistent temperature (25 ± 2 °C), humidity (60 ± 10%), and a 12/12 h light/dark cycle as well as unrestricted access to water and pellet diet. All animal procedures and experimental protocols were conducted in accordance with the Research Ethics Committee for Experimental and Clinical Studies, Faculty of Pharmacy, Cairo University, Cairo, Egypt (IRB number: BC2944). The animals were housed according to the US National Institutes of Health publication Guide for Care and Use of Laboratory Animals (No. 85-23, revised 2011). All attempts were made to reduce the number of animals used and minimize animal suffering.

### 4.2. Drugs and Chemicals

MTX (50 mg/vial) ready for intravenous (i.v.) administration was purchased from Mylan S.A.S., Saint Priest, France. Dexmedetomidine hydrochloride (Precedex™, 200 μg/vial) was purchased from Hospira, Lake Forest, IL, USA. The doses were diluted using sterile water immediately before injection. Leucovorin (LCV, Calcifolinon™, 50 mg/vial) was purchased from GPI, 6th of October City, Egypt.

### 4.3. Experimental Design

**Figure 9:**
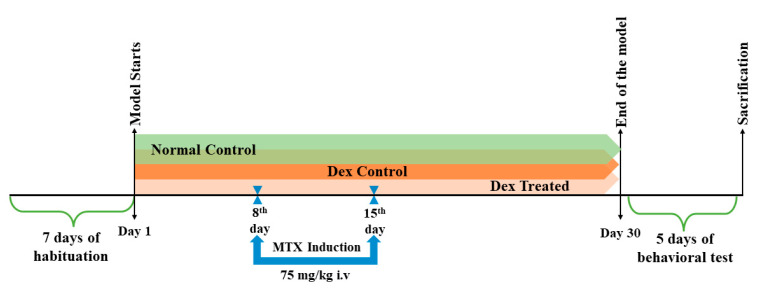
Experimental model timeline. Schematic presentation of the experimental design. Group 1 was administered saline i.p. daily and intravenously on days 8 and 15. Group 2 received 10 μg/kg/day i.p. of Dex for 30 days. Group 3 received MTX at 75 mg/kg intravenously on days 8 and 15, followed by four doses of LCV administered intravenously: 6 mg/kg after 18 h and 3 mg/kg after 26, 42, and 50 h. Group 4 received the same daily dosages of MTX, LCV as in group 3, and Dex as in Group 2.

After 7 days of habituation, the rats were randomly divided into 4 groups (*n* = 15 rats/group). Group size was calculated using power analysis (power = 0.9, α = 0.05) using the G*Power software version 3.1.9.7. Group 1 (normal control): rats were intraperitoneally (i.p.) injected with normal saline daily for 30 days together with i.v. saline on the 8th and 15th days of the model. Group 2 (Dex control): rats received a daily dose of Dex at 10 μg/kg/day i.p. for 30 days [31]. Group 3 (MTX-treated): rats received MTX at 75 mg/kg i.v. on the 8th and 15th days [13,51] followed by four i.p. injections of LCV, the first at a dose of 6 mg/kg after 18 h and the following three at a dose of 3 mg/kg after 26, 42, and 50 h of MTX administration [14,40]. Group 4 (Dex co-treated): rats received a Dex daily dose of 10 μg/kg/day i.p. for 30 days [31] and MTX at 75 mg/kg administered i.v. on days 8 and 15 and LCV as in group 3. The experimental design is displayed in (Figure 9).

**Figure 10:**
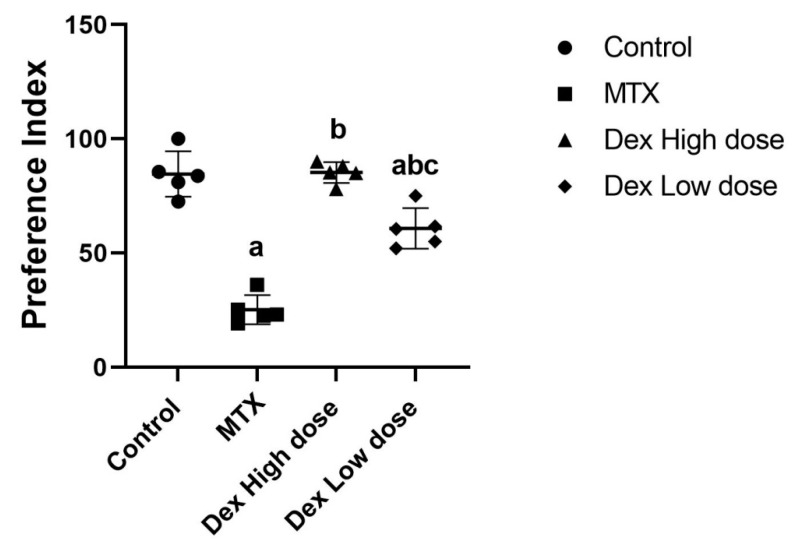
Cognitive and behavioral analysis for the pilot study. Preference index (PI) shows lower preference for the novel object in the MTX group compared to other groups. The pilot study also reveals higher PI in the high dose of Dex co-treatment compared to the low dose of Dex. Data are expressed as mean ± SD, *n* = 5. (a) Significant difference from control, (b) significant difference from MTX, and (c) significant difference from high dose of Dex. Significance level is set at *p* < 0.05. One-way ANOVA followed by Tukey’s post hoc test were used in the statistical analysis.

Notably, we noticed variations in the literature while developing our model: some studies investigating MTX-induced neurotoxicity used LCV after the MTX injection [13,14,40], while others did not [51]. Therefore, we conducted a pilot study, which revealed that the use of LCV in the previously mentioned regimen is necessary to eliminate lethal diarrhea and weight loss resulting from the MTX administration. Our pilot study conclusion was in line with previously published data stating that LCV is important to prevent the lethal effect of high dose MTX without affecting cell proliferation [68]. Due to the high mortality rate observed in the pilot study, we began the model with 15 rats per group to ensure that sufficient samples would be available at the end of the study. Furthermore, our pilot study aimed to examine the difference of the effect between Dex at high and low doses (5 and 10 μg/kg/day i.p. respectively) [31]. The efficacy of the various doses of Dex was assessed using the NOR test in terms of preference index (PI). The results highlighted that the high dose of 10 μg/kg/day i.p. was more efficient than the low dose. The PI in the high dose was found to increase in the co-treated group by 3.38-fold compared to the MTX group while the low dose increased it by 2.4-fold only (*p* < 0.05) (Figure 10).

After 30 days, behavioral assessments were performed to assess the memory and cognitive functions of rats. Twenty-four hours after the behavioral tests, all rats were anesthetized using 3% isoflurane inhalation and sacrificed by cervical dislocation. The brains were dissected and rinsed with ice-cold saline immediately. Four brains from each group were immediately immersed in 10% neutral buffered formalin and transported to the pathology laboratory for histopathology and immunohistochemistry analyses. For the remaining six brains, the hippocampi were dissected from each brain side and stored at −80 °C until the assay of biochemical parameters. All assessments were carried out by blinded investigators.

### 4.4. Behavioral Tests

NOR and MWM were chosen based on their efficacy in testing memory and cognitive function as well as their availability in the animal house and the availability of Any-Maze software for automatic analysis. NOR is a widely used behavioral analysis for spatial memory, studying the short- and long-term spatial memory linked to the hippocampus [69,70]. On the other hand, MWM is used to identify spatial learning and memory in hippocampal affected models [70].

#### 4.4.1. Novel Object Recognition (NOR) Test

NOR was performed at the end of the model to determine the ability of the rats to remember the old object and recognize the novel one. One day before the test, each animal was habituated in an empty open field arena (1 m × 1 m × 0.5 m) for 5 min. On the day of the test, each rat had two trials a familiarization trial and a choice trial which were recorded and analyzed using the ANY-maze software. The arena and the objects were cleaned after each trial with 20% ethanol to remove any olfactory clue.

In the familiarization trial, each rat was introduced to two identical objects. Each rat was allowed to explore the objects for 3 min before being removed from the arena and placed in a separate cage for 15 min. After this period, one of the objects was replaced by a novel object for the choice trial. In the choice trial, each rat was returned to the arena with one old object and another novel object and was allowed to explore them for 3 min before being removed and returned to the normal cage. The exploration time of each object where the rat head was directed to the object in less than 2 cm distance from the object was recorded [40]. The PI was calculated by dividing the time spent investigating the novel object by the total time spent investigating both novel and familiar objects and then multiplying by 100. In addition to the PI, we calculated the DI by dividing the time spent analyzing a novel object minus the time spent examining a familiar object by the time spent examining both novel and familiar objects.

#### 4.4.2. Morris Water Maze (MWM) Test

In this study, MWM test was used to assess the spatial learning of the rats. One day after the NOR, the rats began the training phase of the MWM with 12 training trials in which each rat completed 4 training trials per day for 3 consecutive days before having a probe trial on the fourth day [71]. During the training phase, the platform was placed in the south-east (SE) quadrant of the pool. Four different starting positions were marked, and each rat started a one-minute trial, with four different trials for each position per day, as shown in Table 1. Each trial was limited to a one-minute duration, and the rat was directed to the platform if it did not reach there. The rats were given a rest period of 1 h between the trials. The ANY-mase software was used to record the training and the probe trials. For the MWM, we analyzed various parameters including the escape latency for both the training trials and the probe test as well as the quadrant time and the path efficiency.

### 4.5. Bioinformatic Analysis

To detect the miRNAs that target and regulate the proneuronal transcription factor BDNF, we employed the miRTargetLink 2.0 interactive tool (https://ccb-compute.cs.uni-saarland.de/mirtargetlink2/network/bf99b12a-cd13-453e-bcc1-aa4226ac943e, accessed on 19 Dec 2021). It is a database which includes miRNA, targets, and pathway annotations that help to provide users with a visualization interface to explore and analyze interaction networks between miRNAs and target genes [72]. For the current analysis, we chose to include validated and functional targets. We also used the Human MicroRNA Disease Database (HMDD) (https://www.cuilab.cn/hmdd, accessed on 9 Jan 2022) to detect the association between the selected miRNA and neurological disorders supported by curated experimental evidence [73]. Finally, we employed Pathway Studio (Elsevier, Amsterdam, The Netherlands) (https://www.pathwaystudio.com, accessed on 9 Jan 2022), a freely accessible tool, to analyze the possible associations between miR-15a and other transcription factors that are associated with BDNF and known to be involved in the regulation of the neurogenesis process.

### 4.6. Biochemical Assays

#### 4.6.1. Colorimetric Assay

Tissue homogenization was performed using a cold buffer according to the manufacturer’s instructions and 10% tissue homogenate was prepared. SOD and MDA were assayed using colorimetric kits (Biodignostics^®^, Giza, Egypt, Cat. No: SD 25 21, MD 25 29) according to the manufacturer’s instructions.

#### 4.6.2. Enzyme-Linked Immunosorbent Assay (ELISA)

Rat ELISA kits were purchased to assay the following markers: t-CREB (FineTest^®^, Wuhan, China, Cat. No: ER0914), p-CREB (AFG Bioscience^®^, Northbrook, IL, USA, Cat. No: EK742597), BDNF (Elabscience^®^, Houston, TX, USA, Cat. No: E-EL-R2084), and doublecortin (AFG Bioscience^®^, Northbrook, IL, USA, Cat. No: EK956314). All assays were performed on 10% tissue homogenate using the sandwich ELISA technique according to the manufacturer’s instructions.

#### 4.6.3. Reverse Transcriptase-Quantitative Polymerase Chain Reaction (RT-qPCR)

Total RNA was deduced from the hippocampal tissue lysate using Direct-zol RNA Miniprep Plus (Cat. No. R2072, zymo research corp, Murphy Ave., USA), following the manufacturer’s protocol. The concentration and the purity of each isolated RNA sample were assessed using a Beckman dual spectrophotometer (USA) at 260 and 280 nm. Invitrogen™ SuperScript™ IV One-Step RT-PCR kit (Cat. No. 12594100, Waltham, MA, USA) was used to reverse transcribe the extracted RNA into complementary DNA, followed by real-time PCR amplification of miR-15a-5p and ROCK-1 in a single step according to the manufacturer’s instructions using the Step One Applied Biosystem equipment (Foster city, CA, USA). The sequences of primers were as follows: miR-15a: F: 5′-GCCGAGTAGCAGCACACATAA-3′, R: 5′-CAGTGCGTGTCGTGGAGT-3′, and ROCK-1: F: 5′-AATCTTCCAGTTGGTTCTGCCT-3′, R: 5′-CTCTATTTGGTACAGAAA GCCAACC-3′. Following the RT-qPCR, the results were converted to cycle threshold (Ct.) The Ct values of miR-15a, and ROCK-1 were measured and adjusted to the housekeeping genes, RNU6 and GAPDH, respectively, using the ∆∆Ct method. The primers’ sequences for internal reference genes were as follows: RNU6: F: 5′-GCTTCGGCAGCACATATACTAAA-3′, R: 5′-CGCTTCACGAATTTGCGTGTCAT-3′, and GAPDH: F: 5′-CCTTCTCCATGGTGGTGAAGA-3′, R: 5′-CACCATCTTCCAGGAGCGAG-3′. We calculated the fold change of each gene by taking 2^-∆∆Ct^.

#### 4.6.4. Western Blotting

Protein extraction was performed according to the manufacturer’s instructions using the ReadyPrep^TM^ protein extraction kit from BIO-Rad Inc, Hercules, CA, USA (Cat. No: 1632086). Bradford assay (Bradford Protein Assay Kit (SK3041), BIO basic Inc, Toronto, Canada) was used to assess the protein concentration in each sample according to the manufacturer’s instructions. Each sample with a 20 μg protein concentration was then loaded with an equal volume of 2× Laemmli sample buffer containing 4% SDS, 10% 2-mercaptoethanol, 20% glycerol, 0.004% bromophenol blue, and 0.125 M Tris HCl, with the pH adjusted to 6.8. This mixture was boiled for 5 min at 95 °C to ensure that the protein was denatured prior to loading on polyacrylamide gel electrophoresis.

Polyacrylamide gels were prepared using the TGX Stain-Free^TM^ FastCast^TM^ Acrylamide Kit (SDS-PAGE) (Bio-Rad Laboratories Inc., Cat. No: 161-0181) as directed by the manufacturer. From below to top, the gel was built in a transfer sandwich as follows (filter paper, PVDF membrane, gel, and filter paper), which was placed in a transfer tank containing 1× transfer buffer (25 mM Tris, 190 mM glycine, and 20% methanol). Then, using Bio-Rad Trans-Blot Turbo, the blot was run for 7 min at 25 V to allow protein bands to transfer from the gel to the membrane. At room temperature, the membrane was blocked for 1 h in tris-buffered saline with Tween 20 (TBST) buffer and 3% bovine serum albumin. The blocking buffer had the following components: 20 mM Tris pH 7.5, 150 mM NaCl, 0.1 percent Tween 20, and 3% bovine serum albumin (BSA).

The following antibodies were used for Western blot detection: ERK1/2 (Monoclonal; dilution 1/500, abcam^®^, ab184699) and p-ERK1/2 (Polyclonal; dilution 1/500, abcam^®^, ab214362). At 4 °C, each primary antibody solution was incubated overnight against the blotted target protein. The blot was rinsed 3–5 times with TBST for 5 min. For 1 h at room temperature, the blotted target protein was incubated in the horseradish peroxidase (HRP)-conjugated secondary antibody solution (Goat anti-rabbit IgG- HRP-1 mg Goat mab -Novus Biologicals). The blot was rinsed 3–5 times again with TBST for 5 min. Negative controls were performed without the primary or secondary antibodies. The immunoreactive bands were detected using the chemiluminescence method (chemiluminescent substrate, Clarity^TM^ Western ECL substrate Bio-Rad, Cat. No: 170-5060) according to the manufacturer’s recommendations. A CCD camera-based imager was used to capture the chemiluminescent signals. On the ChemiDoc MP imaging system, the band intensities of the target proteins in each sample were normalized to the protein expression level of β-actin (housekeeping protein) using an image analysis software.

### 4.7. Histology and Immunohistochemistry

Parts of the brain tissues from the rats of different groups were immediately fixed in 10% formol saline for 72 h and processed for preparation of formalin-fixed, paraffin-embedded (FFPE) blocks, and subsequent sectioning. Sections (4 μm) from each paraffin block were stained with hematoxylin and eosin (H&E) for general morphological assessment. Nissl staining was used to detect degraded and intact neurons in the DG and CA3. All standard fixation and staining methods were performed in accordance with the *Handbook of Histopathological and Histochemical Techniques* [74].

Immunohistochemical assays were performed on 5 μm thick sections from the prepared FFPE tissue. Immunohistochemical analysis of Ki-67 expression (Monoclonal, GTX16667, dilution 1/100, GeneTex Co., North America) was carried out. Deparaffinized tissue sections were treated with 0.3% H_2_O_2_ for 20 min and then incubated with the primary antibody overnight at 4 °C. The samples were rinsed and treated with diaminobenzidine for 15 min, washed with a phosphate buffered saline for blocking, dehydrated and clarified in xylene, and cover slipped for microscopic examination.

Six non-overlapping high-power fields were randomly selected and scanned from each sample’s DG areas to determine the area-based percentage of Ki-67 immune-expression levels in the immunohistochemically stained sections [75]. All light microscopic examinations and data collection were performed using the Leica Application module for histological analysis coupled to a full HD microscopic imaging system (Leica Microsystems GmbH, Wetzlar, Germany).

### 4.8. Statistical Analysis

All statistical analyses were performed using the GraphPad Prism 9.0.0 software (121). The data are expressed as mean ± standard deviation (SD). The Shapiro–Wilk and Kolmogorov–Smirnov tests were used to determine whether the variables followed a normal distribution. To compare various groups, one-way ANOVA test was used with Tukey’s post hoc test, except for the training and probe escape latency where two-way ANOVA was used. Dixon’s Q test was used to identify outliers, and Mead’s “Resource Equation” was used to determine whether the sample size was statistically sufficient. For all tests, a *p*-value < 0.05 was considered statistically significant.

## 5. Conclusions

In conclusion, the current study advocates that Dex exerts neuroprotective actions by reversing MTX-induced behavioral, biochemical, and cellular changes in a rat model. The neuroprotective effects of Dex could be partly mediated through modulating the miR-15a/ROCK-1/ERK1/2/CREB/BDNF signaling pathway, reducing MTX-induced oxidative stress, preserving hippocampal neurons, increasing the number of immature neurons, and promoting hippocampal cell proliferation. This study presents Dex as a potential neuroprotective agent that enhances neurogenesis when combined with MTX; however, more compounds can be studied for this purpose, particularly after the molecular mechanism of MTX-induced neurotoxicity has been elucidated.

## Figures and Tables

**Table 1:** Morris water maze starting positions.

Day	Trial 1	Trial 2	Trial 3	Trial 4
**Day 1**	N	W	SW	NE
**Day 2**	SW	N	NE	W
**Day 3**	NE	SW	W	N
**Day 4**	Probe trial started at NW			

N: North, W: West, SW: South-west, NE: North-east, NW: North-west.

## Data Availability

The datasets used and analyzed in the current study are available upon reasonable request from the corresponding author.

## Supplementary Materials

The following supporting information can be downloaded at https://www.mdpi.com/article/10.3390/ijms24010766/s1.

## Abbreviations

**ALL**	Acute lymphoblastic leukemia
**BDNF**	Brain-derived neurotrophic factor
**CA**	Cornu Ammonis
**CRE**	cAMP response element
**CREB**	cAMP-responsive element binding protein
**DCX**	Doublecortin
**Dex**	Dexmedetomidine
**DG**	Dentate gyrus
**DI**	Discrimination Index
**ELISA**	Enzyme-linked immunosorbent essay
**ERK1/2**	Extracellular signal-regulated kinase ½
**FFPE**	Formalin-fixed, paraffin-embedded
**HMDD**	Human microRNA Disease Database
**LCV**	Leucovorin
**MDA**	Malondialdehyde
**miR-15a**	MicroRNA-15a
**MTX**	Methotrexate
**MWM**	Morris water maze
**NOR**	Novel object recognition
**PI**	Preference index
**ROCK-1**	Rho-associated protein kinase 1
**RT-qPCR**	Reverse transcriptase-quantitative polymerase chain reaction
**SGZ**	Subgranular zone
**SOD**	Superoxide dismutase
**SVZ**	Subventricular zone
**TrkB**	Tropomyosin receptor kinase B
