# Supplementary material for: Dexmedetomidine Attenuates Methotrexate-Induced Neurotoxicity and Memory Deficits in Rats through Improving Hippocampal Neurogenesis: The Role of miR-15a/ROCK-1/ERK1/2/CREB/BDNF Pathway Modulation
Source: Int J Mol Sci. 2023 Jan 1;24(1):766. doi: 10.3390/ijms24010766 (PMC9821704; doi:10.3390/ijms24010766)

miRTargetLink 2.0					
**miRNA**	**Target / Target pathway**	**Support**	**Source**	**Experiment(s)**	**Reference - PMID**
hsa-miR-1-3p	BDNF	Functional MTI	MIRT002955	Luciferase reporter assay	14697198
hsa-miR-1-3p	BDNF	Functional MTI	MIRT002955	Luciferase reporter assay	14697198
hsa-miR-1-5p	BDNF	Functional MTI	MIRT732282	Luciferase reporter assay, qRT-PCR, Western blot	27381812
hsa-miR-1-5p	BDNF	Functional MTI	MIRT732282	Luciferase reporter assay, qRT-PCR, Western blot	27381812
hsa-miR-10a-5p	BDNF	Functional MTI	MIRT734858	Luciferase reporter assay, qRT-PCR	28112253
hsa-miR-10a-5p	BDNF	Functional MTI	MIRT734858	Luciferase reporter assay, qRT-PCR	28112253
hsa-miR-124-3p	BDNF	Functional MTI	MIRT000362	LacZ reporter assay	23601049
hsa-miR-124-3p	BDNF	Functional MTI	MIRT000362	LacZ reporter assay	23601049
hsa-miR-132-3p	BDNF	Functional MTI	MIRT054502	qRT-PCR, Western blotting	23704927
hsa-miR-132-3p	BDNF	Functional MTI	MIRT054502	qRT-PCR, Western blotting	23704927
hsa-miR-15a-5p	BDNF	Functional MTI	MIRT734512	Luciferase reporter assay, qRT-PCR, Western blot	26581909
hsa-miR-15a-5p	BDNF	Functional MTI	MIRT734512	Luciferase reporter assay, qRT-PCR, Western blot	26581909
hsa-miR-16-5p	BDNF	Functional MTI	MIRT437463	Luciferase reporter assay, qRT-PCR, Western blot	24026226
hsa-miR-16-5p	BDNF	Functional MTI	MIRT437463	Luciferase reporter assay, qRT-PCR, Western blot	24026226
hsa-miR-182-5p	BDNF	Functional MTI	MIRT054501	qRT-PCR, Western blotting	23704927
hsa-miR-182-5p	BDNF	Functional MTI	MIRT054501	Luciferase reporter assay, qRT-PCR	25955435
hsa-miR-182-5p	BDNF	Functional MTI	MIRT054501	qRT-PCR, Western blotting	23704927
hsa-miR-182-5p	BDNF	Functional MTI	MIRT054501	Luciferase reporter assay, qRT-PCR	25955435

hsa-miR-204-5p	BDNF	Functional MTI	MIRT437447	Immunofluorescence, Luciferase reporter assay, qRT-PCR, Western blot	23285024
hsa-miR-204-5p	BDNF	Functional MTI	MIRT437447	Luciferase reporter assay, qRT-PCR, Western blot	25962115
hsa-miR-204-5p	BDNF	Functional MTI	MIRT437447	Immunofluorescence, Luciferase reporter assay, qRT-PCR, Western blot	23285024
hsa-miR-204-5p	BDNF	Functional MTI	MIRT437447	Luciferase reporter assay, qRT-PCR, Western blot	25962115
hsa-miR-210-3p	BDNF	Functional MTI	MIRT003153	2DGE, immunoprecipitaion, Luciferase reporter assay, Mass spectrometry, Microarray, qRT-PCR, Western blot	19826008
hsa-miR-210-3p	BDNF	Functional MTI	MIRT003153	Luciferase reporter assay, qRT-PCR, Western blot	26708520
hsa-miR-210-3p	BDNF	Functional MTI	MIRT003153	2DGE, immunoprecipitaion, Luciferase reporter assay, Mass spectrometry, Microarray, qRT-PCR, Western blot	19826008
hsa-miR-210-3p	BDNF	Functional MTI	MIRT003153	Luciferase reporter assay, qRT-PCR, Western blot	26708520
hsa-miR-22-3p	BDNF	Functional MTI	MIRT005900	Luciferase reporter assay, Microarray	21168126
hsa-miR-22-3p	BDNF	Functional MTI	MIRT005900	Luciferase reporter assay, Microarray	21168126
hsa-miR-30a-5p	BDNF	Functional MTI	MIRT001946	Luciferase reporter assay	18632683
hsa-miR-30a-5p	BDNF	Functional MTI	MIRT001946	Luciferase reporter assay	18632683
hsa-miR-613	BDNF	Functional MTI	MIRT735486	Luciferase reporter assay, qRT-PCR, Western blot	27545218

hsa-miR-613	BDNF	Functional MTI	MIRT735486	Luciferase reporter assay, qRT-PCR, Western blot	27545218
hsa-miR-96-5p	BDNF	Functional MTI	MIRT734161	Luciferase reporter assay, qRT-PCR	25955435

Immunohistochemistry Pictures

A) Hematoxylin and Eosin (H&E) Staining

I. DG

i. Normal Control

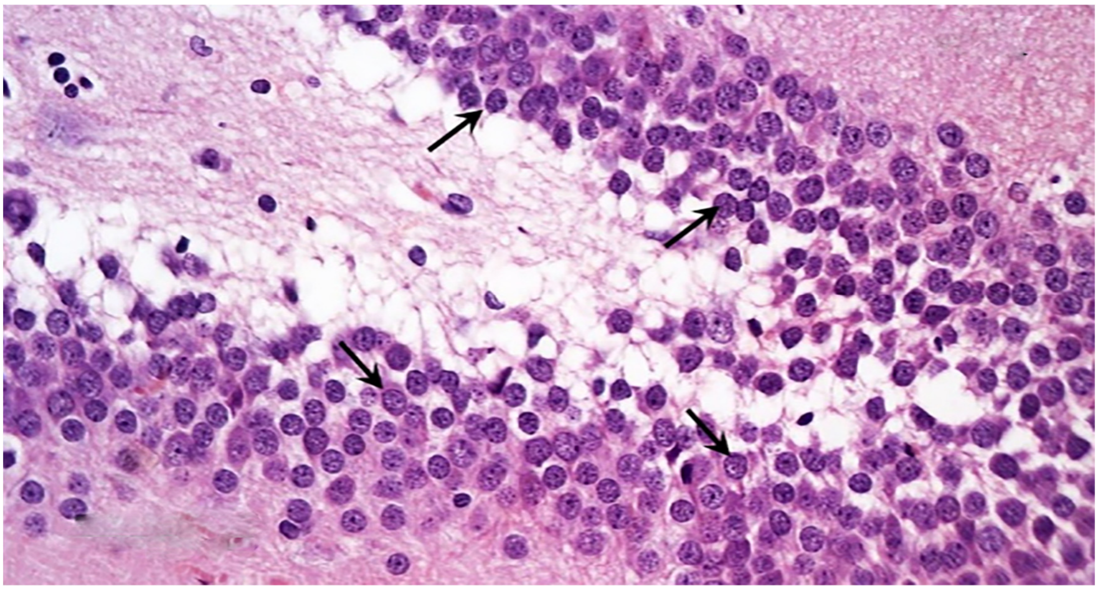

ii. Dex Control

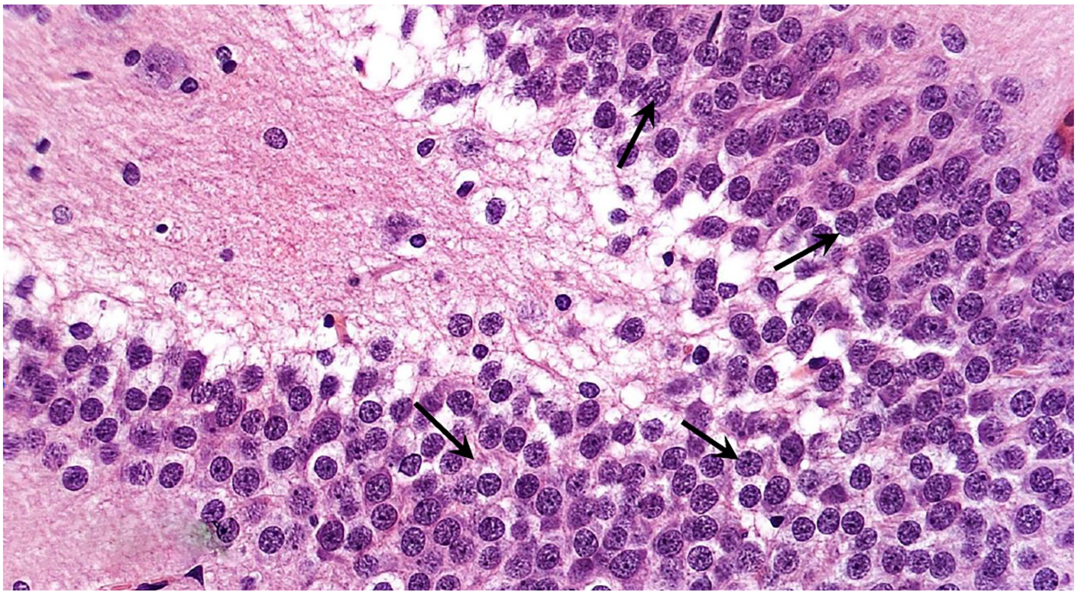

iii. MTX

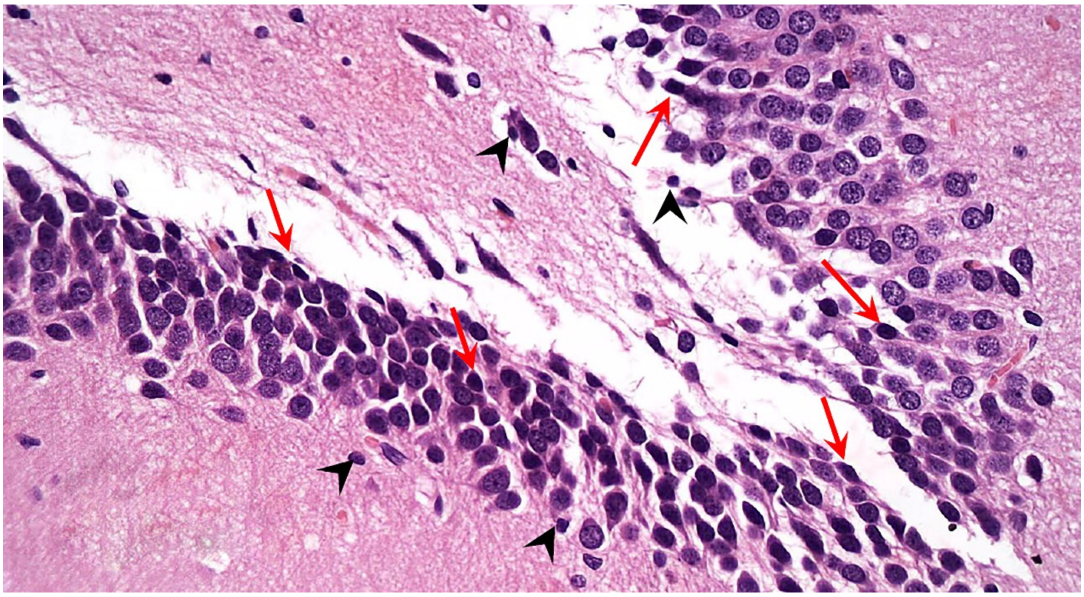

iv. Dex Co-treated

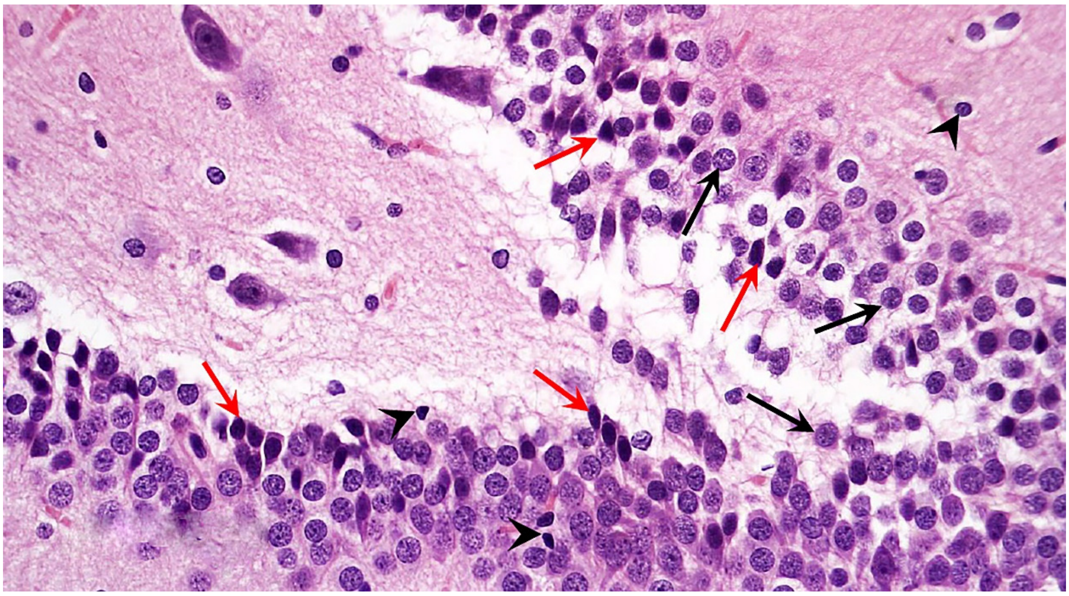

II. CA3

i. Normal Control

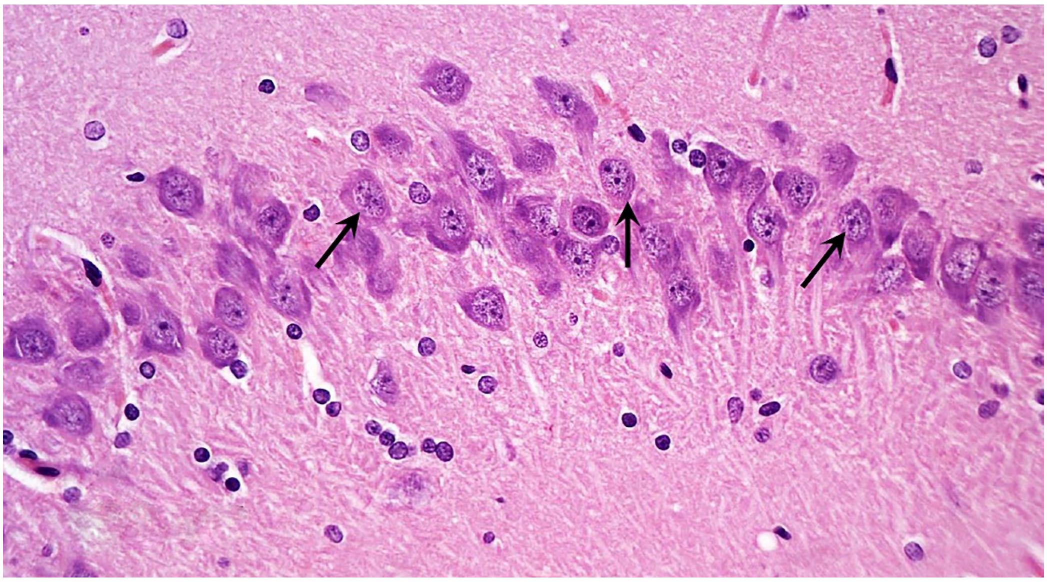

ii. Dex Control

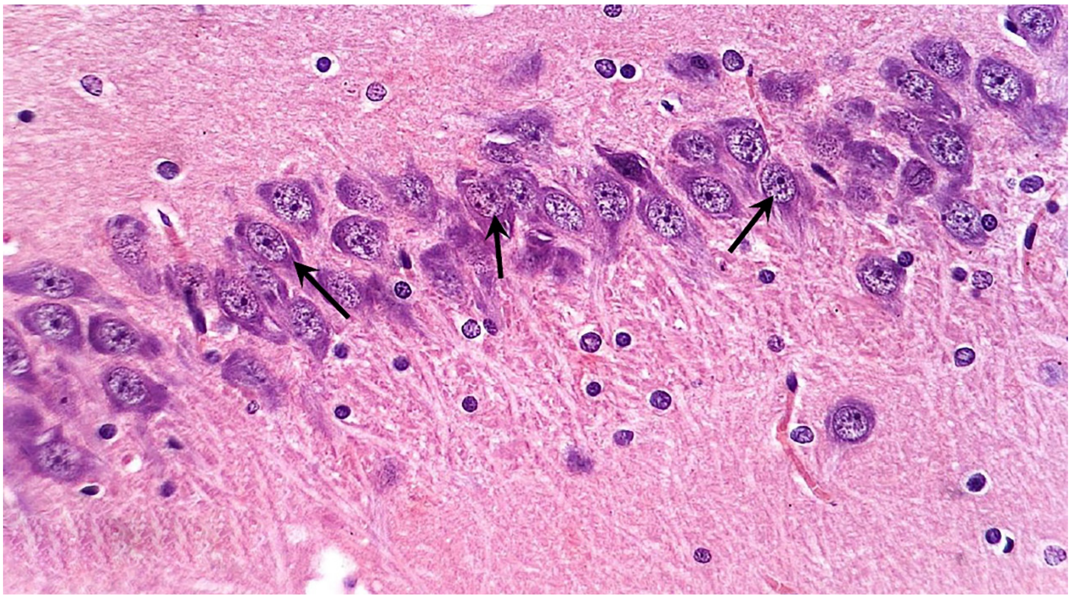

iii. **MTX**

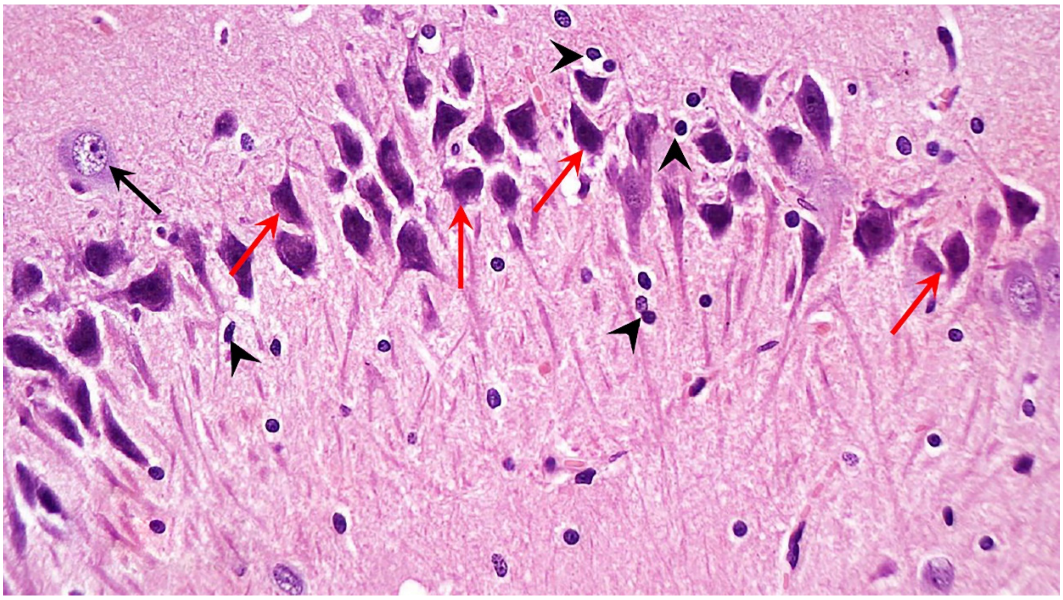

iv. **Dex Co-treated**

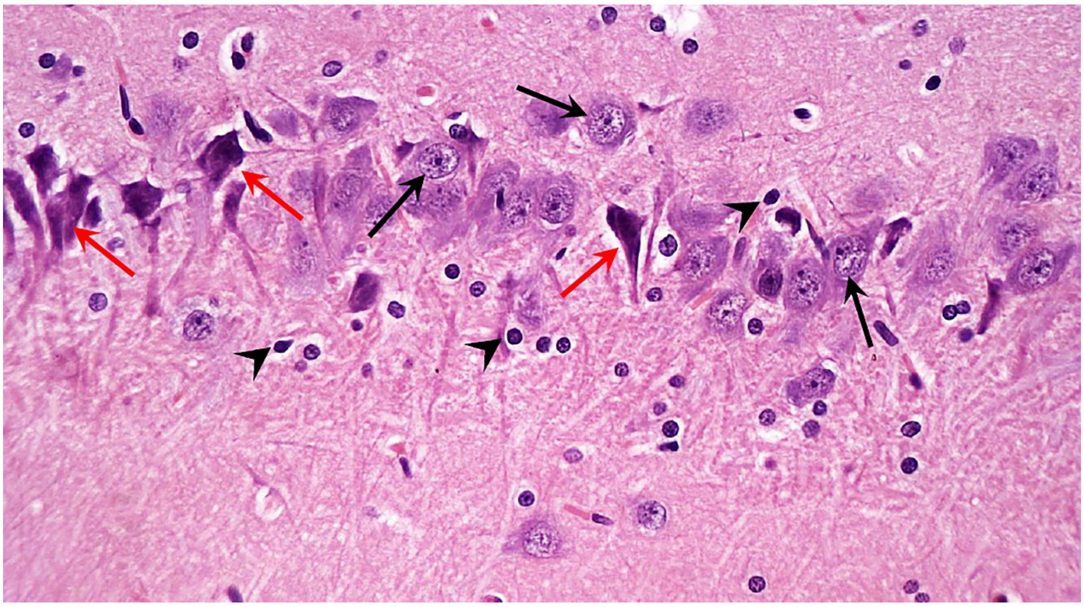

B) Nissl Staining

I. DG

i. Normal Control

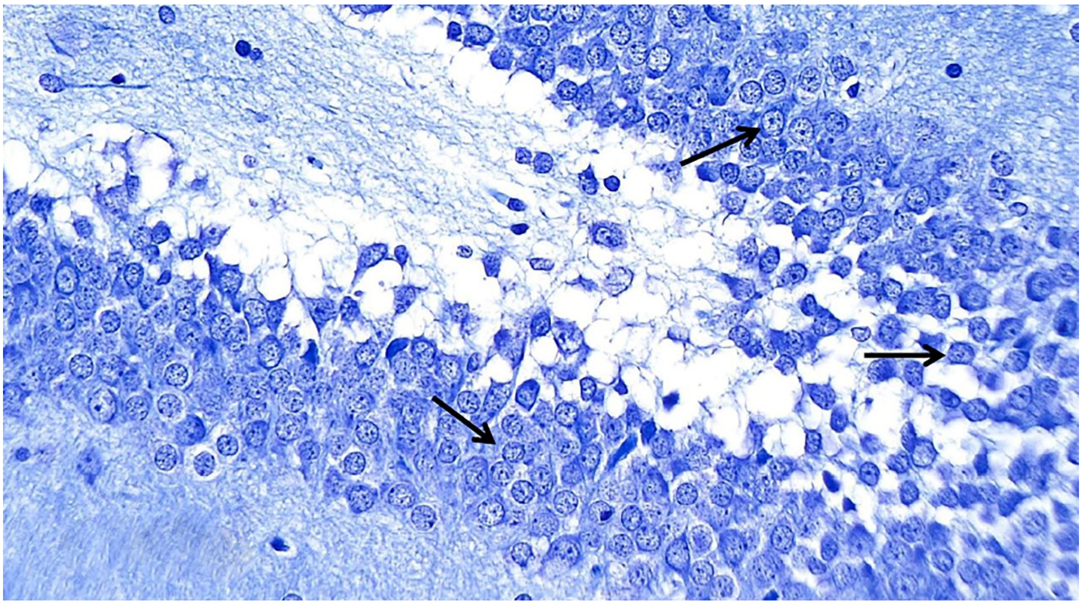

ii. Dex Control

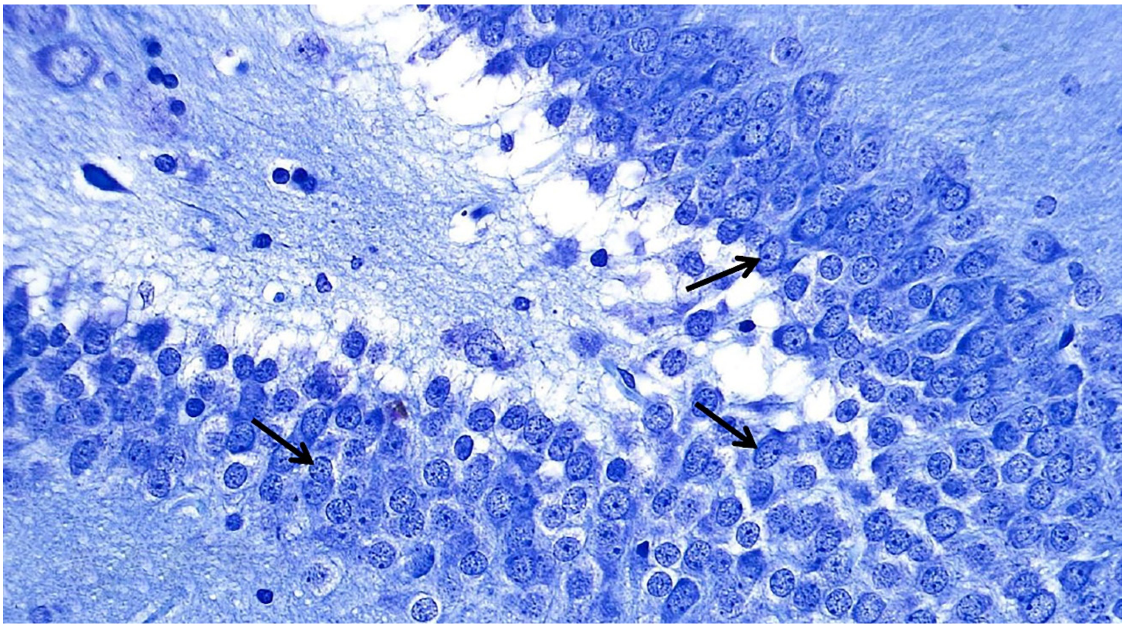

iii. MTX

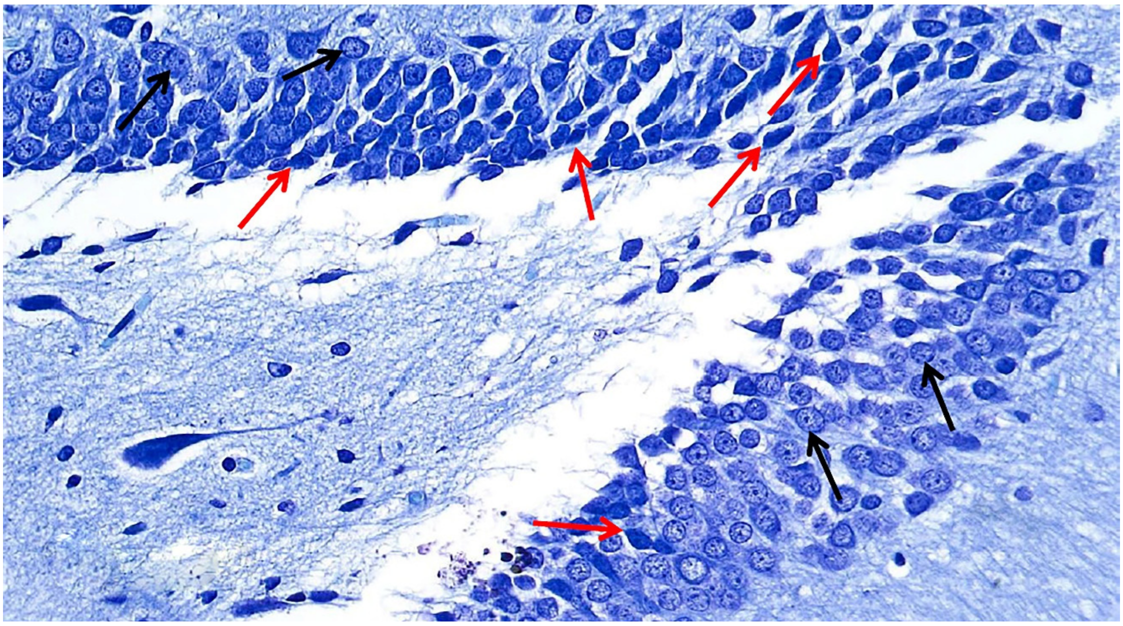

iv. Dex Co-treated

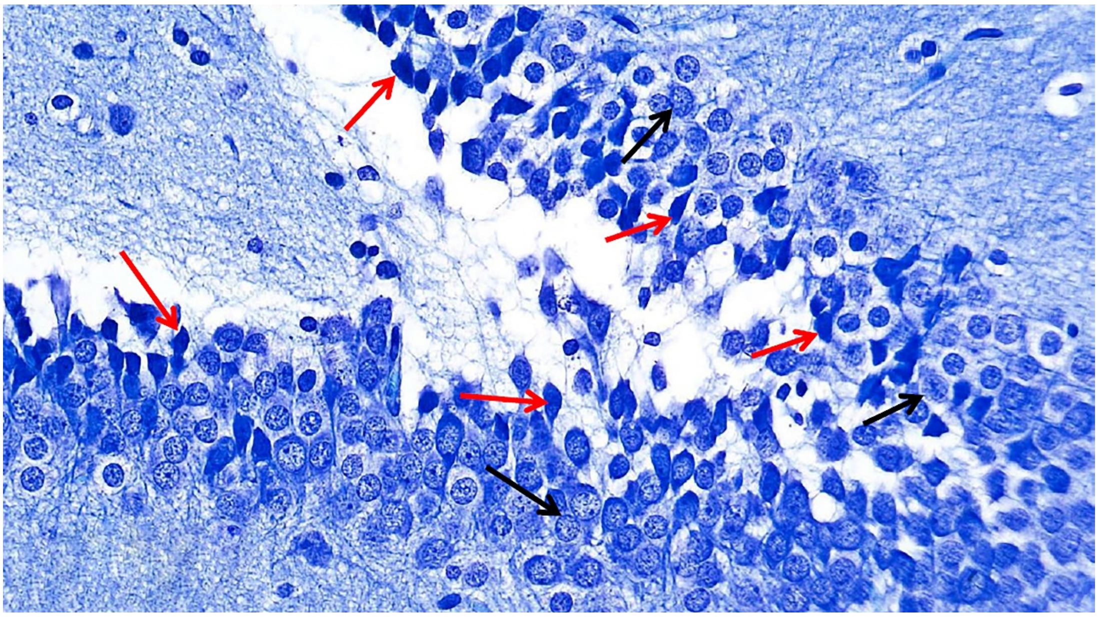

II. CA3

i. Normal Control

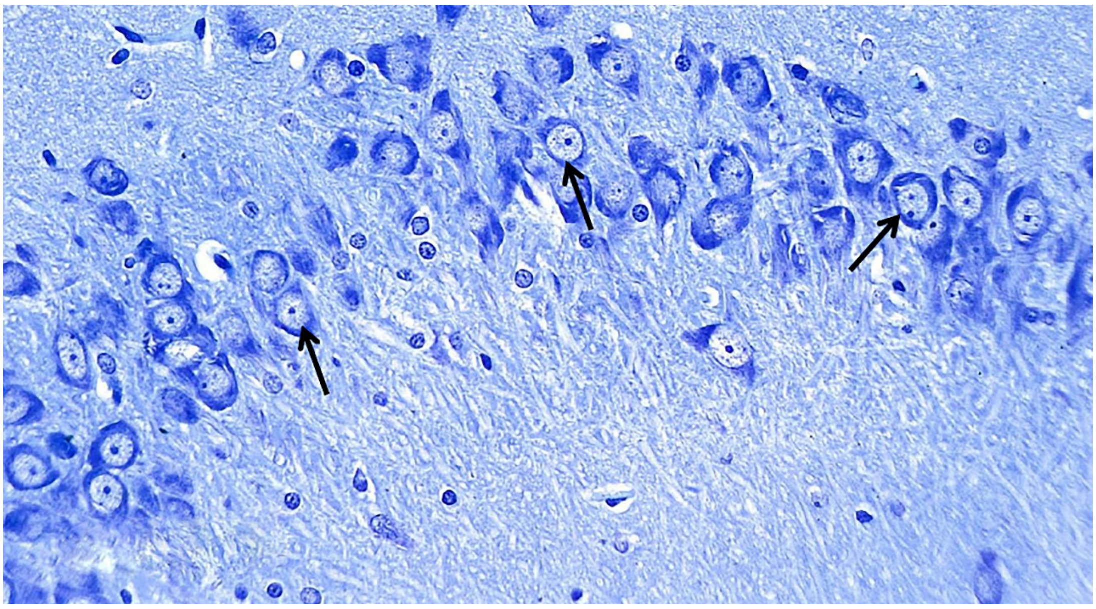

ii. Dex Control

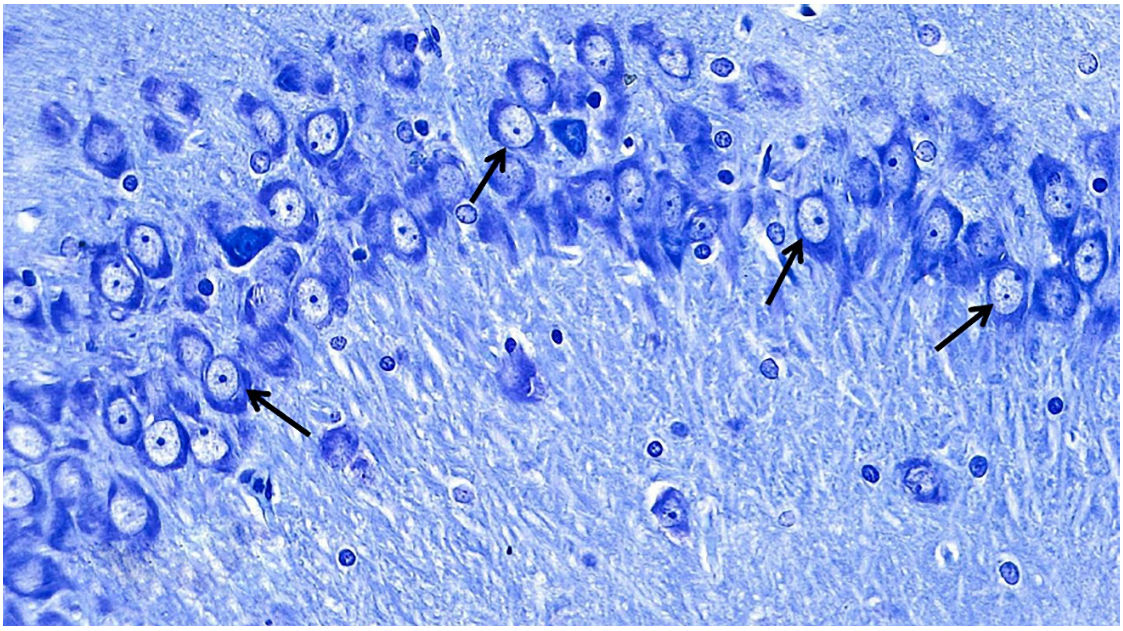

iii. MTX

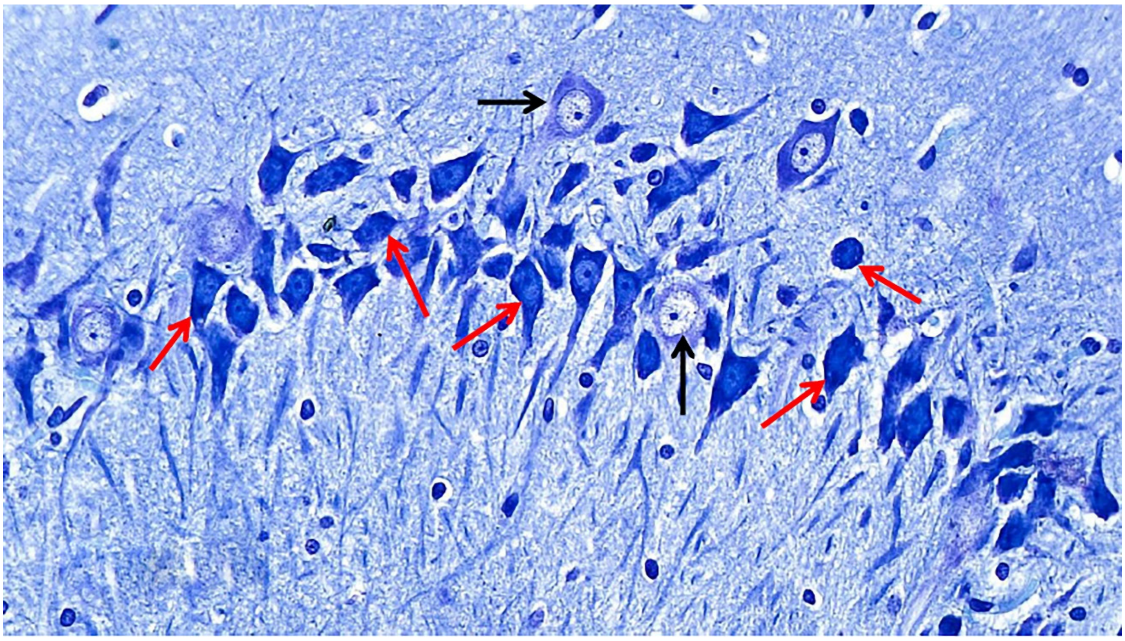

iv. Dex Co-treated

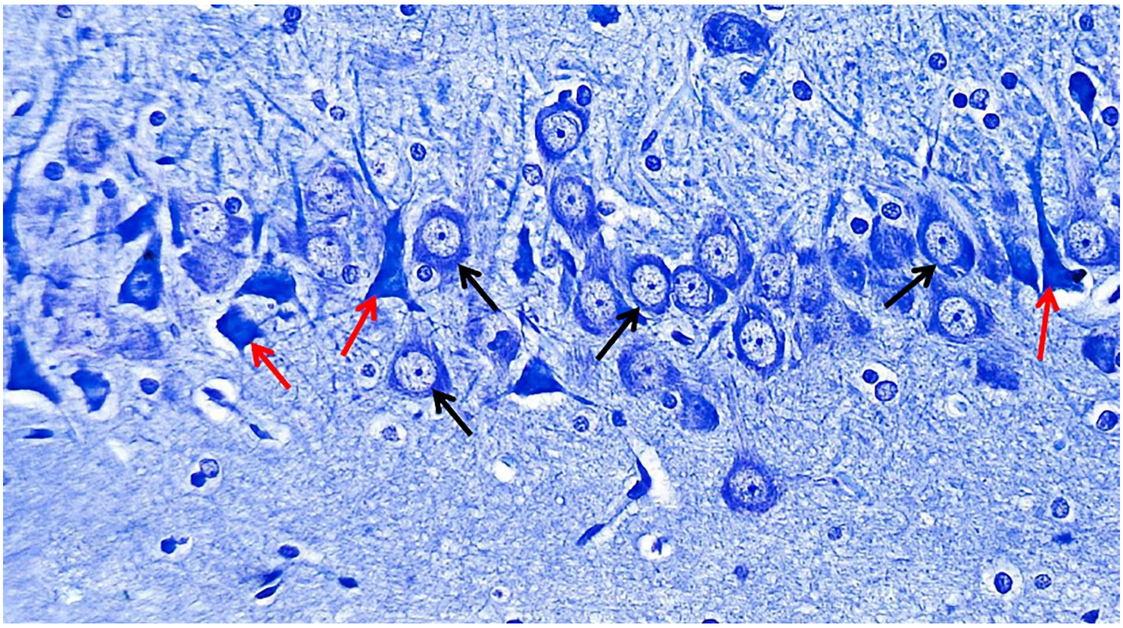

C) Ki-67

i. **Normal Control**

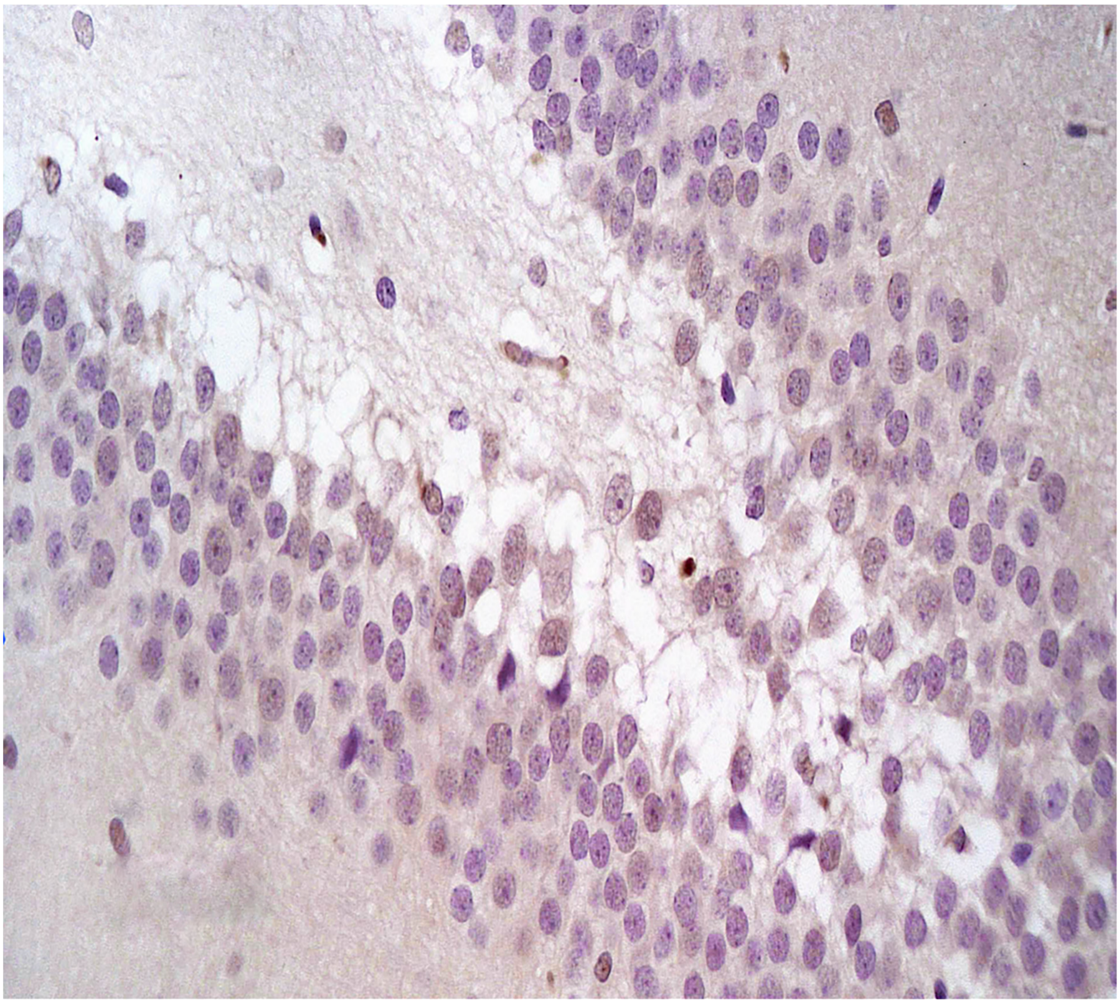

ii. **Dex Control**

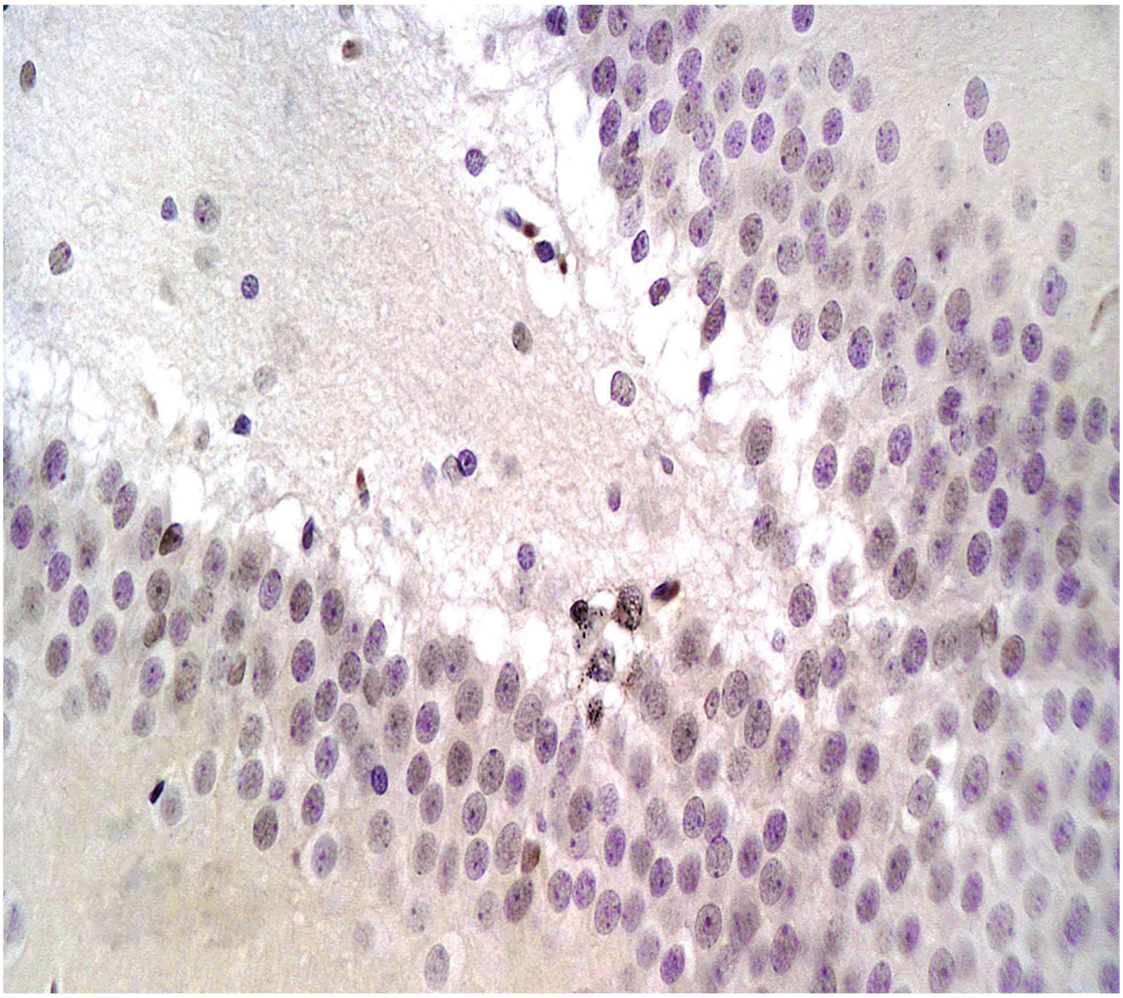

iii. MTX

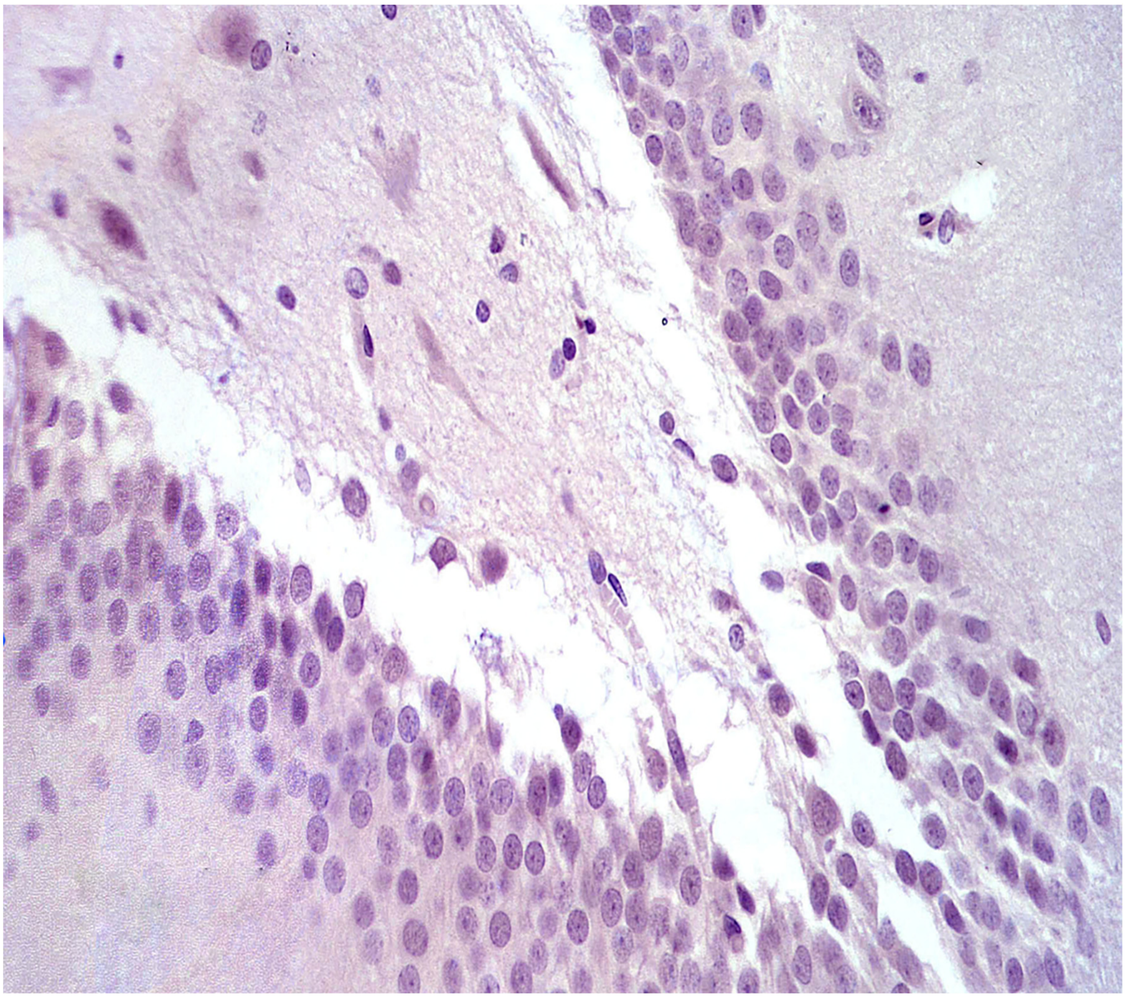

iv. Dex Co-treated

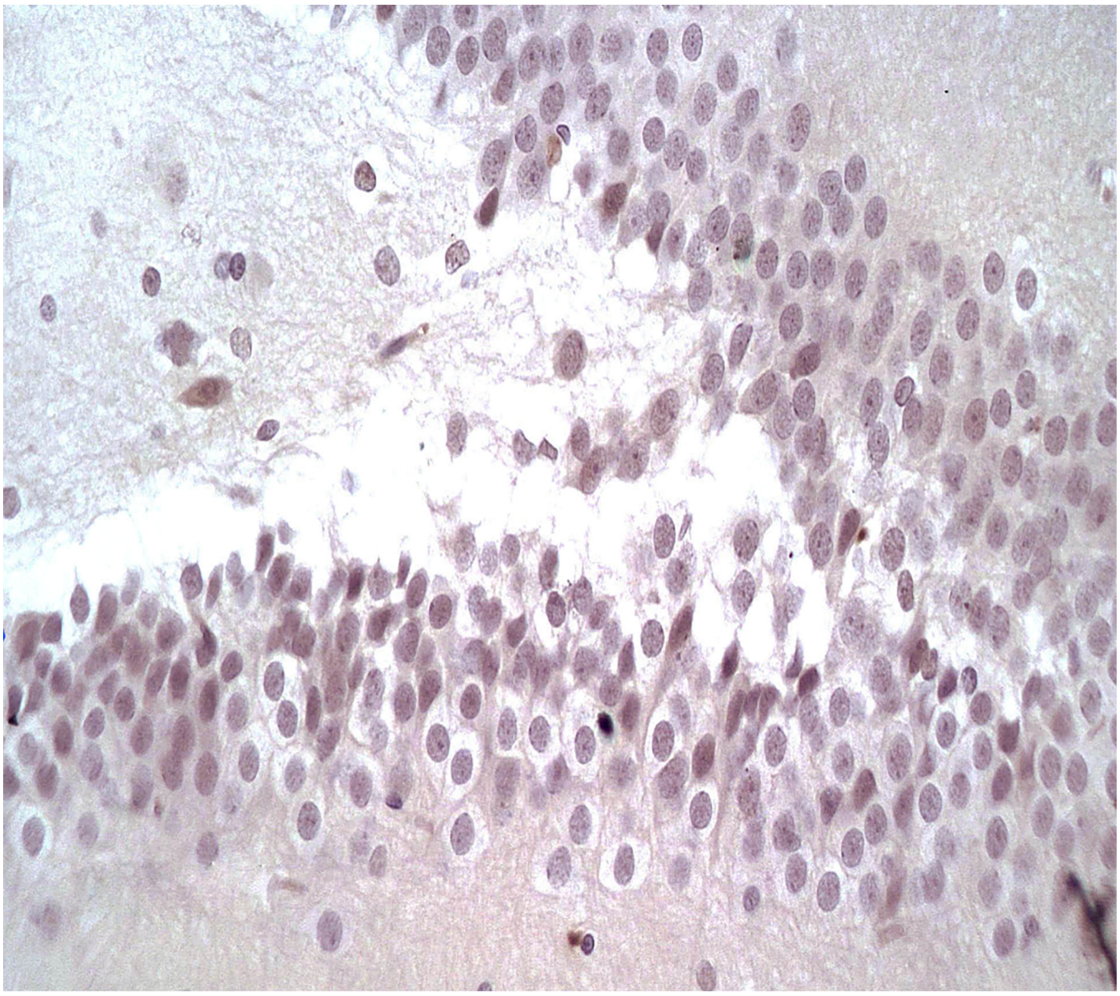

Supplement: Supplementary file 1 [file ijms-24-00766-s001.zip › ijms-2106892-supplementary.pdf]
